# Essential gene disruptions reveal complex relationships between phenotypic robustness, pleiotropy, and fitness

**DOI:** 10.15252/msb.20145264

**Published:** 2015-01-21

**Authors:** Christopher R Bauer, Shuang Li, Mark L Siegal

**Affiliations:** Department of Biology, NYU Center for Genomics and Systems BiologyNew York, NY, USA

**Keywords:** heterogeneity, pleiotropy, robustness, variation

## Abstract

The concept of robustness in biology has gained much attention recently, but a mechanistic understanding of how genetic networks regulate phenotypic variation has remained elusive. One approach to understand the genetic architecture of variability has been to analyze dispensable gene deletions in model organisms; however, the most important genes cannot be deleted. Here, we have utilized two systems in yeast whereby essential genes have been altered to reduce expression. Using high-throughput microscopy and image analysis, we have characterized a large number of morphological phenotypes, and their associated variation, for the majority of essential genes in yeast. Our results indicate that phenotypic robustness is more highly dependent upon the expression of essential genes than on the presence of dispensable genes. Morphological robustness appears to be a general property of a genotype that is closely related to pleiotropy. While the fitness profile across a range of expression levels is idiosyncratic to each gene, the global pattern indicates that there is a window in which phenotypic variation can be released before fitness effects are observable.

## Introduction

Measurement of any quantitative trait in any population will return a distribution of values. The standard reductionist approach to biology, the prevalence of population-based molecular assays, and the relative abundance of mean-centric statistical tests have directed the vast majority of biological inquiry toward understanding the factors that influence phenotype means. Meanwhile, the dispersions of values around the mean and the shapes of the distributions have remained largely invisible or ignored (Geiler-Samerotte *et al*, 2013). Although the traditional approach of studying population means has proven fruitful in many situations, a growing body of evidence suggests that our understanding of many basic biological processes looks very different at the level of individuals and the phenomena that contribute to, or are contingent upon, differences between individuals have not received adequate attention (Cai *et al*, 2008; Eldar *et al*, 2009; Sharma *et al*, 2010; Aldridge *et al*, 2012; Trott *et al*, 2012).

Phenotypic differences between individuals are derived from three sources: genetic variation, differences in environments, and stochastic processes. Genetic variation is generally the most appreciated and, in many cases, the most well understood of these factors. It is also common knowledge that environmental conditions play a critical role in shaping the ultimate phenotypes of organisms. However, it has only been in the past decade that the role of stochastic processes in phenotypic expression has gained much attention (Raser & O'Shea, 2004; Eldar & Elowitz, 2010). Progress has been hindered not only by technological challenges in monitoring individual cells and molecules, but also by intellectual barriers. Whereas genetic and environmental factors can have easily observable effects on phenotype mean values, the primary impact of stochastic events is on the variance associated with a given phenotype. Thinking about variances is still somewhat foreign to many biologists and requires new intellectual frameworks. Nonetheless, there is a growing appreciation of the importance of randomness in biology (Raj *et al*, 2010; Levin *et al*, 2011).

It has long been appreciated that there is a need to limit fluctuations within a cell, but phenotypic heterogeneity can play a beneficial role. In order to produce tissues that comprise a mixture of cell types, many developmental processes have harnessed stochastic processes to facilitate differentiation. Color vision requires multiple types of photoreceptor cells that express different pigments. During development, chance interactions between enhancers and promoters can cause photoreceptor cells to express a single pigment gene (Jacobs, 2009; Johnston & Desplan, 2010). Heterogeneity in olfactory receptors seems to be established by similar mechanisms (Lomvardas *et al*, 2006).

Within microbial populations, epigenetic switching provides a constant source of phenotypic heterogeneity that can help a subset of cells to survive stressful conditions and take advantage of chance opportunities in an unpredictable environment (Balaban *et al*, 2004; Thattai & van Oudenaarden, 2004; Kussell & Leibler, 2005). This variability has implications for human disease since it can affect microbial drug resistance and pathogenicity (Soll, 1997; Balaban *et al*, 2004). Moreover, these same concepts are relevant to cancer. Recent work has shown that reversible transitions between chromatin states generate heterogeneity in aggressive melanoma tumors. This provides subpopulations of cells with enhanced abilities to metastasize or survive drug treatments (Roesch *et al*, 2010; Sharma *et al*, 2010). Understanding the biological mechanisms that underlie phenotypic heterogeneity will be critical to develop better treatment options to combat metastases and recurrence of cancers.

Perhaps the most promising model for studying these aspects of biology is the yeast, *Saccharomyces cerevisiae*. In addition to the wealth of tools and data already available, yeast appear to display some similar properties to melanoma cells with regard to heterogeneity and reversible phenotypic switching. While most individual yeast cells proliferate rapidly, a small fraction exhibit greatly reduced growth rates. These slow growing cells, marked by high levels of TSL1 protein, exhibit increased stress resistance and are able to produce offspring that revert back to the regime of rapid growth (Levy *et al*, 2012). Exploring the molecular mechanisms underlying these phenomena may provide unique insights that can ultimately be applied to diseases such as cancer.

Yeast are also highly amenable to genome scale, systematic screens. This has provided a glimpse into the genetic basis of phenotypic heterogeneity. In a massive undertaking, nearly all nonessential gene knockout strains were stained, imaged, and scored for a huge number of morphological phenotypes (Ohya *et al*, 2005). These data have not only provided an important resource for the yeast community, but a unique opportunity to assess the contribution of genes to the phenotypes of single cells.

Using these data, we had previously identified ∽300 nonessential yeast genes that reduce the effects of stochastic or microenvironmental variation on the expression of cellular morphology (Levy & Siegal, 2008). That is, genetically identical cells in the same environment showed significantly greater morphological variation in the absence of these genes than in their presence. These gene products are termed phenotypic stabilizers (Masel & Siegal, 2009). The identified phenotypic stabilizers tend to participate in core cellular processes and tend to be highly connected within genetic or protein–protein interaction networks (Levy & Siegal, 2008). Essential genes also tend to participate in core cellular processes and tend to be highly connected in cellular networks to an even greater extent than the nonessential phenotypic stabilizers. These observations raise the hypothesis that essential genes may play the primary role in modulating the robustness of phenotypes to stochastic and microenvironmental variation (Levy & Siegal, 2008).

We had also previously proposed, based on simulations of evolving gene-regulatory networks, that the suppression of phenotypic variation is an intrinsic property of complex genetic networks and that deletion of any gene in such a network is likely to increase variation (Siegal & Bergman, 2002; Bergman & Siegal, 2003). It has also been proposed that genetic perturbations may increase phenotypic variation due to a general impairment of cellular networks that is associated with a reduction in fitness. This was evidenced by a negative relationship between growth rate and phenotypic potential, a measure of the extent to which a mutation increases phenotypic variation, within the set of nonessential knockout strains (Wang *et al*, 2011).

In this study, we have expanded our analysis of phenotypic stabilization to include the majority of essential yeast genes by taking advantage of a collection of hypomorphic mutants. Each strain in this collection has been engineered with an altered 3′ UTR to reduce the expression of a single essential gene by a method called decreased abundance by mRNA perturbation (DAmP) (Breslow *et al*, 2008). For each of 873 haploid DAmP strains, we have collected images of more than 1,000 individual cells. We analyzed these images using CalMorph (Ohya *et al*, 2005) to measure more than 100 morphological phenotypes in each strain. Our analyses indicate that essential gene perturbations are far more likely to result in increased phenotypic heterogeneity than are nonessential gene deletions. The release of previously constrained variation appears to be a property that is specific to individual genes and cannot be explained simply by gene product dosage. The stabilization of morphological variation by a given gene can be generalized across many phenotypes even after accounting for correlated morphological features. The increased heterogeneity within a specific genetic background is strongly linked to its degree of pleiotropy (the extent to which a mutation alters multiple, independent phenotypes). However, the increased heterogeneity is not often predictive of the fitness of that genotype, affording the possibility that such variation may have evolutionary relevance.

## Results

### Phenotypic stabilization is a common function of essential genes

**Figure 1:**
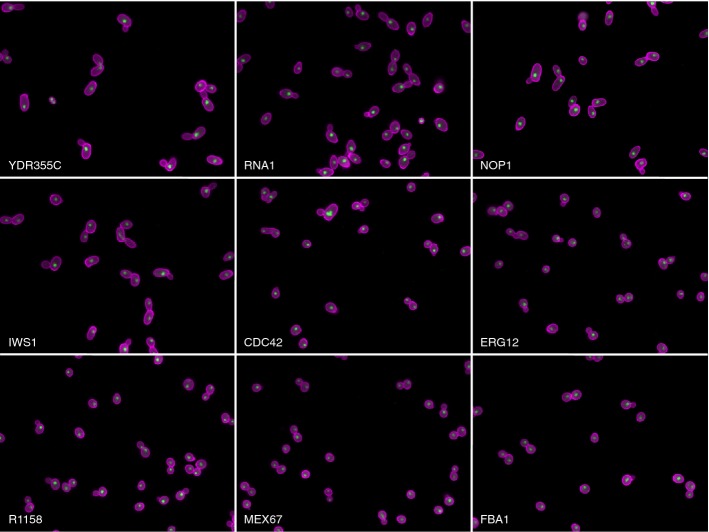
Example images of mutants with high or low levels of phenotypic variation Cell walls are labeled with FITC-conjugated concanavalin A (magenta) and nuclei are labeled with DAPI (green). DAmP-YDR355C (phenotypic potential = 3.25), DAmP-RNA1 (phenotypic potential = 1.81), DAmP-NOP1 (phenotypic potential = 3.07), DAmP-IWS1 (phenotypic potential = 2.14), DAmP-CDC42 (phenotypic potential = 2.54), and DAmP-ERG12 (phenotypic potential = 1.61) are all high-confidence phenotypic stabilizers. R1158 (phenotypic potential = −0.05) does not contain any essential gene mutations. DAmP-MEX67 (phenotypic potential = −1.42) and DAmP-FBA1 (phenotypic potential = −1.01) are DAmP strains that display low phenotypic variation.

We used high-throughput fluorescence microscopy to collect images of cells from 873 strains in the haploid DAmP collection. Each strain in this collection contains an insertion in the 3′ UTR of a single essential gene. These insertions destabilize the corresponding mRNA and typically cause a reduction of 2- to 10-fold in protein levels compared to wild-type cells (Breslow *et al*, 2008). We stained the cells with FITC-conjugated concanavalin A to mark the cell wall and DAPI to mark the nucleus (see Materials and Methods) (Fig1). We then imaged the cells and processed the images using CalMorph to obtain measurements of 187 phenotypes (Ohya *et al*, 2005). For each strain, at least three biological replicates were performed, yielding data for at least 1,000 cells per strain. In total, we analyzed over 2 million individual cells.

We summarized the total amount of phenotypic variation within a strain by a measure called phenotypic potential (Levy & Siegal, 2008; Costanzo *et al*, 2010; Lehner, 2010; Venancio *et al*, 2010; Wang *et al*, 2011; Graham *et al*, 2012; Simko & Csermely, 2013; Yvert *et al*, 2013). Our method for calculating phenotypic potential (detailed in Materials and Methods) is similar to that described previously (Levy & Siegal, 2008) with two major exceptions. First, the presence of multiple replicates for each genotype allowed us to use mixed-effect linear modeling to estimate the effect of individual preparations on each phenotype measurement. In this way, we were able to identify 15 phenotypes (mostly related to fluorescence intensity) that were highly variable between replicates and exclude them from further analyses. We also excluded an additional 40 phenotypes for other reasons such as those with multimodal distributions that are not well characterized by standard measures of dispersion or phenotypes with frequent missing values (see Materials and Methods), leaving 132 high-quality phenotypes. Second, we used a different approach to deal with non-independence of the phenotypes measured by CalMorph. The original analysis was based on the summary statistics (mean and standard deviation) of each genotype rather than individual cell measurements and relied upon partitioning around medoids on mean-corrected standard deviations to select largely independent phenotypes for subsequent analysis (Levy & Siegal, 2008). Here, we performed principal component analysis on the individual cell measurements. This approach allowed us to combine a group of highly correlated phenotypes into a single, more precise measure rather than to discard all but one of them. For downstream analyses, we considered only the top 41 principal components that retain >90% of the total variance present in the 132 high-quality CalMorph phenotypes (Supplementary Figs S8, S9 and S10).

The 41 principal components, henceforth referred to simply as phenotypes, actually comprise three groups, because CalMorph classifies cells into three classes prior to measurement (A: unbudded, B: budded with one nucleus, C: budded with two nuclei). Because principal component analysis was performed on each class separately, some between-genotype correlations between phenotypes from the different classes remain. Nevertheless, the 41 phenotypes represent morphological measurements that are not appreciably correlated with each other. We next calculated the standard deviation of each phenotype for each strain. These raw standard deviations cannot be used directly as measures of phenotypic variation, for two reasons. First, across all genotypes, the observed standard deviations can have a strong dependence on means. We therefore used the residuals from loess regressions of standard deviations against means to generate mean-corrected measures of variation for each phenotype (Levy & Siegal, 2008; Geiler-Samerotte *et al*, 2013). Second, if two or more random variables are correlated, the standard deviation of their first principal component will be larger than the standard deviations of any of the original variables. Since we did not want to weight our analysis toward phenotypes that were measured redundantly, we normalized the residuals such that each principal component had a standard deviation equal to one.

A final phenotypic potential score was then defined as previously (Levy & Siegal, 2008): a strain's normalized residual standard deviations were ranked across phenotypes and the top half (20 phenotypes in this case) were averaged. Because a large number of phenotypes are averaged, this phenotypic potential score captures the extent to which a mutation increases general morphological variation, rather than variation specific to a particular phenotype (Levy & Siegal, 2008). Averaging the ∽50% most-variable phenotypes, rather than all of them, reduces noise in the score (Levy & Siegal, 2008). The number of phenotypes averaged had very little effect on the determination of which genes are phenotypic stabilizers (Supplementary Fig S1).

**Figure 2:**
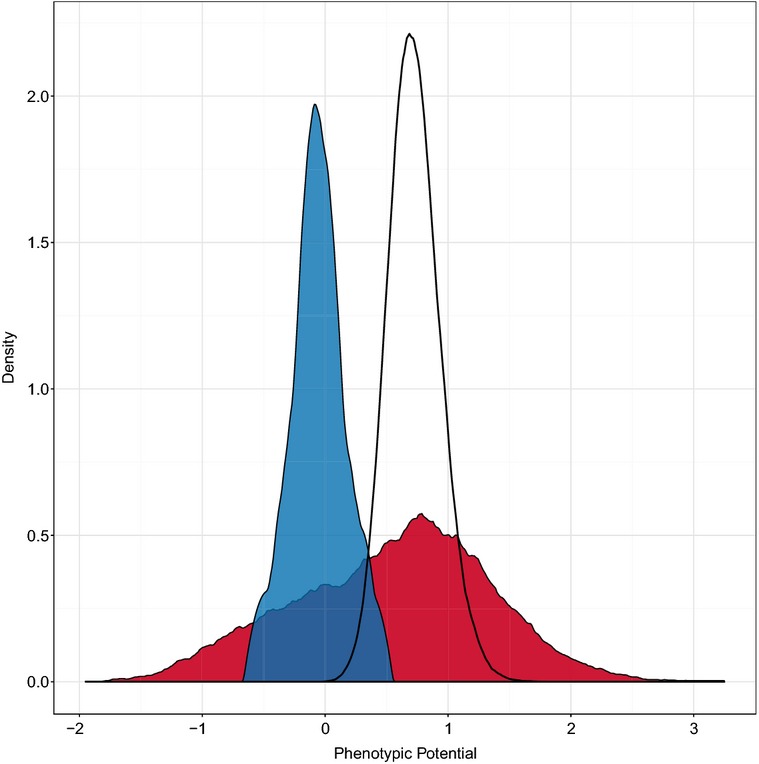
Distribution of phenotypic potential scores A kernel density plot of the phenotypic potential scores from all DAmP strains is shown in red. Random permutation of the same dataset yields the probability density of phenotypic potentials shown as the black curve. In blue is the distribution of phenotypic potentials from a wild-type control strain.

We next sought to define the set of genes that functioned as phenotypic stabilizers by determining a threshold above which phenotypic potential scores were unlikely to fall by chance. Randomly permuting our entire dataset to break associations between cells and genotypes allowed us to empirically estimate the probability of observing a given phenotypic potential score by chance and thereby to estimate the false discovery rate (FDR) given our data. The maximum number of true positives is expected to occur at a phenotypic potential threshold of 1.065, which corresponds to a FDR of 0.06. Using this threshold, we estimate that at least 175 essential genes (20% of those assayed) function as phenotypic stabilizers (Fig2). This percentage is considerably higher than that estimated for nonessential genes, 7.1% (Levy & Siegal, 2008).

Our method for calculating phenotypic potential is expected to give a conservative estimate of the percentage of phenotypic stabilizers. The loess regression assumes that the bulk of the mutants do not exhibit increased phenotypic variation. The more phenotypic stabilizers there are contributing to a dataset, the more the residuals from the loess regression are compressed downward (Levy & Siegal, 2008). Given that a large percentage of DAmP strains nonetheless had significantly high phenotypic potential values, it is likely that the actual percentage of phenotypic stabilizers among the essential genes is much higher. To better assess this, we estimated an alternative null expectation based on the parental strain that the DAmP library was derived from. We performed 12 biological replicates of R1158 and added the resulting phenotypic data to our dataset. The mean phenotypic potential score of the replicates was −0.05 ± 0.21. Allowing three standard deviations from the mean phenotypic potential for the reference strain, we estimate that 455 essential genes (52% of those assayed) function as phenotypic stabilizers (Fig2). Even this may be an underestimate given that some of the DAmP strains may not reduce expression enough to have substantial phenotypic effects.

**Figure 3:**
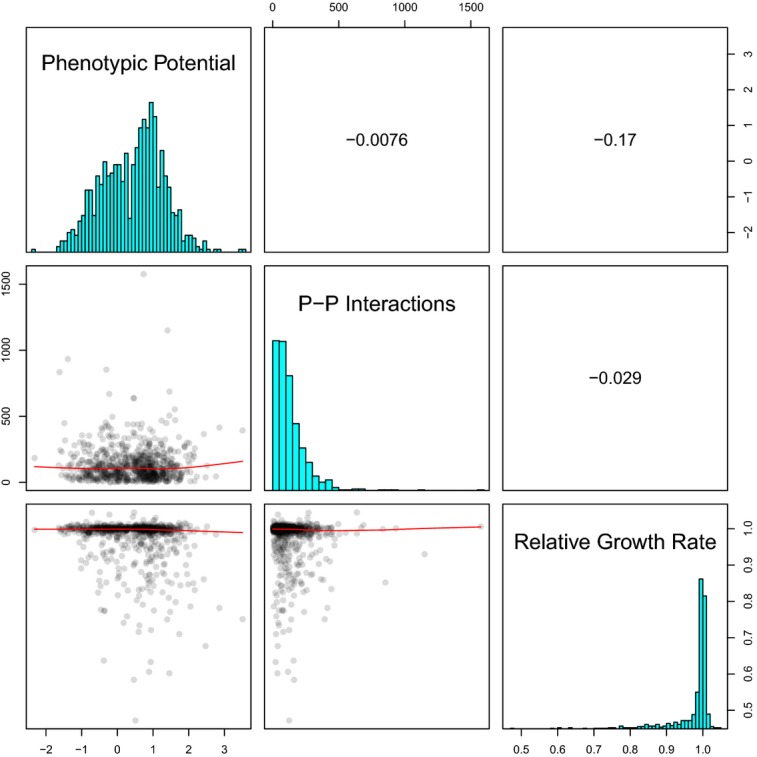
Correlations between phenotypic potential, the number of protein–protein interactions from BioGRID, and the published relative growth rates The lower-left panels show the pairs of variables plotted against each other with the red lines indicating loess regressions. The diagonal panels display the underlying distributions of each metric. The upper right panels show the Pearson correlation coefficients for each comparison.

Regardless of the threshold used, it appears that the essential genes are highly enriched for phenotypic stabilizers compared to the nonessential genes, as predicted. Recall that a basis for this prediction was that, for nonessential genes the number of protein–protein interactions correlates positively with phenotypic potential, and essential genes have on average even more protein–protein interactions than nonessential phenotypic stabilizers (Levy & Siegal, 2008). We therefore examined whether phenotypic potential correlates with the number of protein–protein interactions among the essential genes represented in the DAmP collection; however, we did not observe a significant correlation (Fig3). For a subset of the DAmP collection, genetic interaction data are available, but again there was no relationship between the number of genetic interactions and phenotypic potential (Supplementary Fig S4). We postulate that the strong tendency of essential genes to have very large numbers of interactions limits the possibility of finding further enrichment within the subset of essential genes with high phenotypic potential.

Similarly, nonessential phenotypic stabilizers are enriched for genes annotated to core cellular processes (Levy & Siegal, 2008), so we tested for enrichment of gene ontology (GO) terms among the essential phenotypic stabilizers. We were unable to find any GO terms that were enriched among the phenotypic stabilizers relative to the entire set of genes in the DAmP collection. This is not completely surprising, given that the genes in the DAmP collection are already very highly enriched for a large number of terms.

### Robustness can be generalized across phenotype space

Our measure of phenotypic potential summarizes phenotypic heterogeneity, within a given genotype, across a high-dimensional phenotypic space. We thus wished to explore further how this heterogeneity was distributed across individual phenotypes. In one extreme possibility, perturbing a gene could result in a massively increased variation for a very small number of phenotypes without any effect on—or even a reduction in—the variation of others. At the other extreme, a gene perturbation could increase the standard deviation associated with many independent phenotypes. This can be thought of as pleiotropy in variation. Our data suggest that the latter scenario is closer to the truth for most genes.

The first approach we took was simply to vary the number of phenotypes that were averaged to calculate phenotypic potential and to compare the resulting scores. Even when we compare the two most extreme scenarios, defining phenotypic potential either as the normalized residual standard deviation associated with the single most-variable phenotype or as the average of the variances for all 41 phenotypes, the rank order of the genes remains similar (Spearman's ρ = 0.73) (Supplementary Fig S1). If we average the standard deviations of at least 10 phenotypes, the rank order of genes quickly converges (Spearman's ρ > 0.88 for all pairwise comparisons) (Supplementary Fig S1).

Thus, if a given genotype displays a large residual standard deviation in almost any single phenotype, it will tend to display substantial variation across many morphological phenotypes. This result is surprising given that the phenotypes we are comparing are principal components that are, by definition, uncorrelated within each cell class of our full dataset. Trivial explanations of this result, such as geometrical constraints or other morphological patterns that are common to all yeast cells, are therefore precluded. Rather, these findings indicate that the robustness of cell morphology can be thought of as a general feature of a given genotype, at least with regard to single-gene perturbations. Recent analyses of wild yeast isolates with a wide range of genetic diversity suggest that this conclusion may be applicable more broadly, as a subset of isolates showed high variance for many independent cell-morphology phenotypes (Yvert *et al*, 2013).

**Figure 4:**
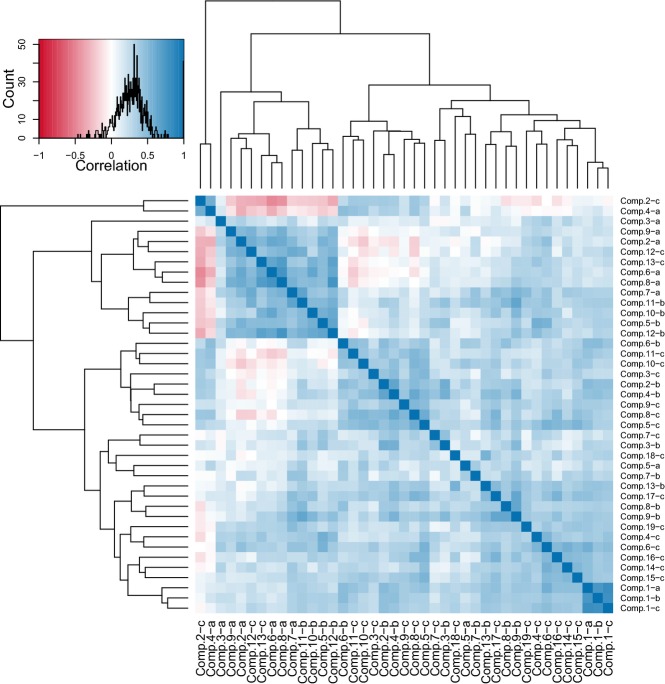
Pairwise correlations between phenotype standard deviations Each strain in the DAmP collection was assigned a mean adjusted standard deviation for each of the top 41 principal components. The distribution of correlation coefficient values between the standard deviations of each pair of phenotypes, across all strains, is shown in the upper left panel. The intensity of the blue shading in the heat map indicates the degree of positive correlation, while that of red shading indicates the degree of negative correlation. The dark blue cluster in the bottom right corner are the first principal components from unbudded (a) small budded (b) and large budded (c) cell categories, which all correlate strongly with cell size. Components 2-c and 4-a are measures of the position of the nucleus within the cell and are the only two phenotypes with standard deviations that tend to correlate negatively with other phenotype standard deviations. The dendrograms were determined by hierarchical clustering.

We next sought to identify any phenotypes that broke with this trend of general morphological robustness. An analysis of the pair-wise correlations between residual phenotype standard deviations across all genotypes indicated that nearly all showed a positive relationship. It is worth noting that the average correlation between all phenotype mean pairs is negligible at 0.01 (Supplementary Fig S5). Across all phenotype pairs, the average correlation of the residual variances was 0.27. Only 15 comparisons (∽1% of the total) had Pearson's correlation coefficients less than −0.2 (Fig4). This subset was dominated by two phenotypes, principal components 2C and 4A (Fig4). Both of these components are composed solely of CalMorph phenotypes that relate the position of the nucleus to other cellular landmarks. That is, genotypes with high variability in nuclear position within the cell tend to have relatively low variability in other phenotypes, and vice versa. It is difficult to speculate what biological significance this pattern may have, if any.

### Pleiotropy is strongly correlated with phenotypic potential

Since the phenotypic potential associated with a genotype summarizes variability across many independent phenotypes, we hypothesized that this measure might be related to pleiotropy. In other words, mutations that increase variation in multiple phenotypes may also alter multiple phenotypic means. It is not necessarily the case that this hypothesis should be true. As previously described, we used the residuals from loess regression of phenotype standard deviations against their respective means as our ultimate measures of dispersion for calculating phenotypic potential. This approach controls for instances where the mean value of a particular phenotype has any systematic relationship to the standard deviation.

As a measure of pleiotropy, we simply counted the number of phenotype means that differed from the wild-type reference. Given the large number of cells in each genotype, most of the DAmP strains showed statistically significant changes to most phenotypes. Thus, we required that the mean value for a mutant must fall beyond one standard deviation of the wild-type mean in order to be counted as having an effect.

There are several definitions of pleiotropy, which can lead to confusion (Paaby & Rockman, 2013). It is therefore important to specify precisely what type of pleiotropy is being considered. Our pleiotropy score directly measures what has been termed “developmental pleiotropy”, or the extent to which a mutation affects multiple, independent traits (Paaby & Rockman, 2013). Less directly, it may also measure what has been termed “molecular gene pleiotropy”, or the number of functions a gene has (Paaby & Rockman, 2013).

**Figure 5:**
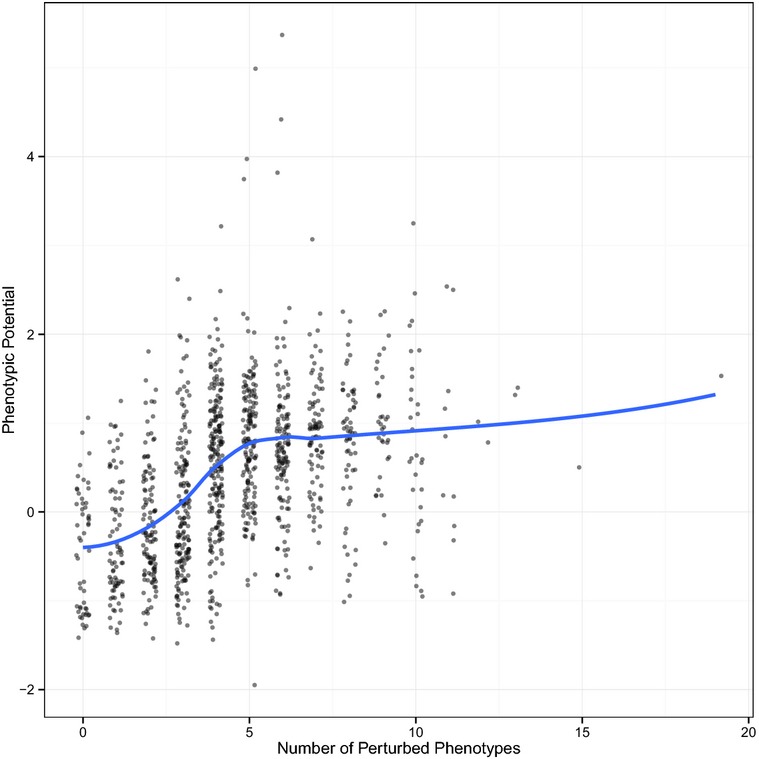
Relationship between pleiotropy and phenotypic potential For each DAmP strain, the number of phenotypes that differ from the wild-type reference by at least one standard deviation are plotted on the *x*-axis. A small amount of noise was added to prevent overplotting. Phenotypic potential scores are plotted along the *y*-axis. The blue line indicates the result of a loess regression.

A plot of the number of affected phenotypes versus phenotypic potential reveals a strong positive relationship with Spearman's ρ = 0.48 (Fig5). Loess regression shows that this relationship is nearly linear up to approximately 4 affected phenotypes and tapers off with additional phenotypes. While we could not directly detect over-representation of genetic or physical interactions among the strongest stabilizers, pleiotropy may be a proxy for network centrality. Previous analyses of nonessential gene knockouts has suggested that hubs in genetic networks tend to be enriched for (molecular gene) pleiotropic effects (Costanzo *et al*, 2010) as well as for effects on phenotypic variation (Levy & Siegal, 2008). The finding that a relationship between pleiotropy and variation holds in a completely independent dataset provides strong support to the idea that hubs in biological networks not only impact mean values of multiple phenotypes but also play a primary role in limiting the effects of stochastic variation.

### Phenotypic robustness is not predictive of fitness

We next wished to determine whether the release of phenotypic variation, upon gene perturbation, could be simply a side effect of general cellular impairment and loss of fitness, as has been proposed (Wang *et al*, 2011). To test this hypothesis, we measured fitness-related phenotypes and compared these measurements to our phenotypic potential scores.

**Figure 6:**
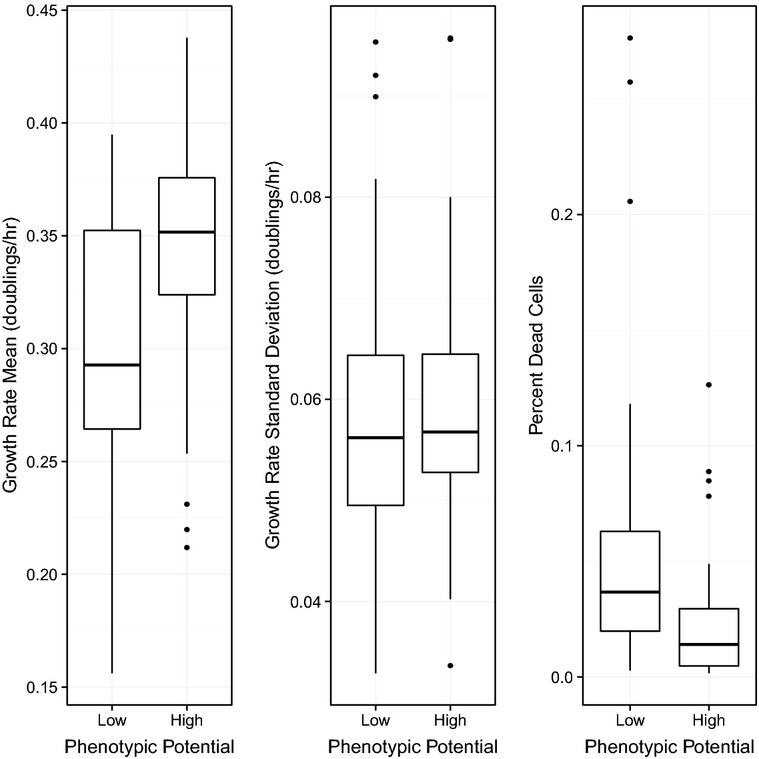
Relationship between phenotypic potential and fitness Within each panel, the 48 DAmP strains with the lowest phenotypic potentials were grouped on the left and compared to the 48 DAmP strains with the highest phenotypic potentials, grouped on the right. Microcolony-based measurement of mean growth rate is plotted in the left panel. Standard deviation of growth rate is plotted in the middle panel. The percentage of dead cells in the population is plotted in the right panel.

We first focused our investigations on subsets of strains at the extremes of the phenotypic potential distribution, where differences would be easiest to observe. The 48 DAmP strains with the highest phenotypic potentials were named Hi-Var, and the 48 lowest were called Low-Var. Each of these strains was subjected to individual microcolony growth-rate measurements as described previously (Levy *et al*, 2012). In brief, single cells were deposited on glass-bottom, 96-well plates in liquid media and colony areas were tracked by time-lapse microscopy for 9 h. For each genotype, specific growth rates (doublings in microcolony area per hour) for approximately 5,000 microcolonies were obtained. When we compared phenotypic potential to mean growth rate, we surprisingly observed a weak positive correlation (*R* = 0.28) (Fig6). The average specific growth rate of the Hi-Var strains was 0.345 doublings/h compared to 0.299 doublings/h for the Low-Var strains. Although this result is statistically significant (*P* < 0.0003), it is clear that mean growth rate is not a strong predictor of variability in morphology. This result is not easily reconciled with the hypothesis that high phenotypic potential is generally caused by low fitness.

One of the major advantages of performing growth-rate analysis on individual microcolonies that are derived from single cells is that the variance in growth rates within a population can be measured along with the mean (Levy *et al*, 2012; Ziv *et al*, 2013). Given that the extent of variation is highly correlated across most morphological phenotypes, we suspected that the extent of variation in growth rates might also correlate. This was not the case. Comparing phenotypic potential to the standard deviation in growth rate resulted in an extremely poor correlation (*R* < 0.01) (Fig6). Substituting the growth-rate coefficient of variation for the standard deviation produced a better correlation (*R* = 0.25); however, this is still a weaker relationship than with mean growth rate alone indicating that the dispersion in growth rate did not add any additional predictive power. Thus, it seems that morphological robustness is not necessarily linked to robustness in non-morphological phenotypes. Perhaps this is due to fundamental differences in the selection pressures that have shaped variation of these traits.

One remaining possible explanation for the lack of correlation between morphological variation and growth rate is that many of the strains with relatively high growth rates might also have increased death rates relative to wild-type. If this were true, the variability in morphological phenotypes might be inflated by a modest fraction of cells that were near death and had extreme morphologies. To test this possibility, we added 1 μg/ml propidium iodide (a marker of dead cells) to the growth medium and repeated our growth-rate analysis with the Hi- and Low-Var strains. Immediately after plating the cells, we collected bright-field and fluorescence images of all fields followed by time-lapse tracking in bright field only. Those cells with fluorescent nuclei at the first time point were counted as dead, while all others were considered to be alive, and thus the proportion of dead cells could be calculated for each strain. When we compared phenotypic potential to the proportion of dead cells, again there was no appreciable association (*R* = 0.15; Fig6).

In addition to our own measurements of the growth rates of the Hi-Var and Low-Var strains, we compared phenotypic potential scores for each of the DAmP strains with their growth rates relative to wild-type, which had been measured previously by competition assays (Breslow *et al*, 2008). A simple linear regression with phenotypic potential as a function of relative growth rate revealed a very weak negative association *R* = −0.17 (Fig3). This relationship was similar to, but slightly weaker than, that found between growth rate and phenotypic potential in the nonessential-gene knockout collection (Wang *et al*, 2011). It is puzzling to note that when we compared the published relative growth rates of the DAmP strains with the 96 strains that we measured by the microcolony assay, there were substantial inconsistencies (Supplementary Fig S2). Measurements of bulk growth rates by optical density also show the same inconsistencies (Supplementary Fig S3). Surprisingly, the vast majority of the published growth rates for the DAmP strains are very close to wild-type (more than 1 in 4 are reported to out-compete wild-type cells) yet we measured a wide range of growth-rate defects for all of these strains. This discrepancy is difficult to resolve as the batch-competition method used to determine the relative growth rates should be extremely sensitive.

### Phenotypic stabilization is gene specific and dosage dependent

One caveat of our analysis is that each DAmP mutation has a unique degree of “knockdown” and the extent to which each mutation affects the molecular processes that the gene participates in is largely unknown. It is possible that the genes we identified as stabilizers simply correspond to the strains where the gene dosage was reduced enough to see a phenotype. If this were true, our false-negative rate would be high and it would indicate that the vast majority of essential genes can function as phenotypic stabilizers. To assess this possibility, we utilized an independent, tunable method to perturb essential genes (Mnaimneh *et al*, 2004). In this “Tet” system, each essential gene has a constitutively active promoter whose transcriptional activity can be down-regulated to various degrees by the addition of doxycycline. In this way, the gene's expression can be reduced to a desired level by adjusting the concentration of doxycycline in the growth medium (Mnaimneh *et al*, 2004).

To examine the effects of specific gene expression levels on morphological variation, we selected a set of 34 candidate genes to conduct an in-depth analysis. The doxycycline-repressible strains we chose all had reported growth rates near wild-type levels in YPD and all had reported severe growth defects, or complete lack of growth, in the presence of 10 μg/ml doxycycline (Mnaimneh *et al*, 2004). These genes also spanned nearly the entire range of phenotypic potential values in the DAmP collection. We grew each strain to saturation in parallel cultures of YPD media containing 16 different concentrations of doxycycline ranging from 0 to 20 μg/ml, since most of the strains failed to grow at measureable rates within this range. These cultures were then diluted 1:100 into YPD with the same concentration of doxycycline as before, grown for 8 h, and prepared for imaging identically to the DAmP strains. We also included 7 biological replicates of the ancestor strain that does not respond to doxycycline to insure that most cells in the dataset were not affected. Each of these strains was also analyzed using a microcolony growth assay to determine its growth rate (Levy *et al*, 2012).

We first sought to address the extent of the false negatives from the DAmP data. For 112 conditions that contained the wild-type strain, R1158, the phenotypic potential scores ranged from −1.20 to 0.36 with a mean of −0.43. Surprisingly, every Tet strain showed a phenotypic potential higher than 0.36 in at least one condition and 75% of all strain by doxycycline combinations fell above this level. Though the range phenotypic potentials varied greatly by gene, it appeared that every gene we tested played at least a limited role in buffering phenotypic variation. It is important to note that unlike the DAmP system, the Tet responsive alleles are not under the control of their native regulatory sequences and thus may be overexpressed at low concentrations of doxycycline and any complex expression dynamics will be abrogated. It is also reasonable to assume that the Tet system can alter the levels of gene expression noise due to transcriptional bursting. This would provide a clear mechanism of how cell-to-cell variation could be established. Regardless, these data demonstrate that variation in morphological phenotypes is exquisitely sensitive to proper expression of essential genes.

**Figure 7:**
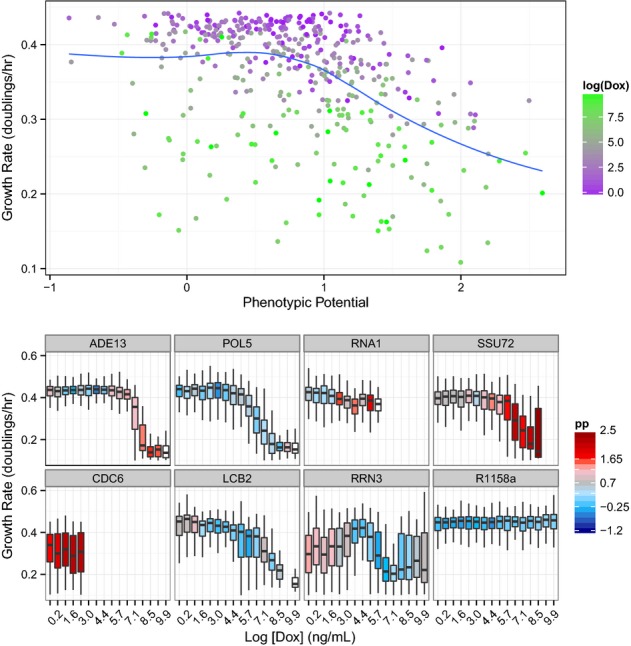
Phenotypic variation and growth rates in Tet-repressible strains In the top panel, all phenotypic potential scores are plotted against microcolony growth rates for all Tet strains across all concentrations of doxycycline. The points are shaded to indicate the concentration of doxycycline ranging from zero (purple) to 20 μg/ml (green). The lower panels show the dynamics of eight individual genes. In each case, the levels of doxycycline range from zero on the far left to 20 μg/ml on the far right. Box plots show the distributions of growth rates observed with color shading to indicate the observed phenotypic potential (blue = low variation, red = high variation, white boxes indicate too few cells to calculate a phenotypic potential, and the absence of a box indicates fewer than 10 colonies observed).

We next sought to use this system to independently address the relationships between pleiotropy, growth rate, and phenotypic potential. A comparison between the number of phenotypes that differ from wild-type against phenotypic potential using the Tet system yields results that are very similar to those found from the DAmP alleles. While less pleiotropy was observed in general, there remains a strong positive correlation between the two measures for 0–5 phenotypes, after which the relationship breaks down (Supplementary Fig S11). Pooling all of the data together, it is also apparent that there is a negative correlation between growth rate and phenotypic potential in this context. Specifically, this correlation is only apparent when phenotypic potential is greater than one, and for values less than one, there is little if any trend (Fig7).

Individual examination of each gene reveals a much wider range of behaviors (Supplementary Figs S6 and S7). Five of the genes tested (ARP4, CDC42, CDC6, POL1, and POL30) exhibit elevated phenotypic potentials even in the absence of doxycycline and showed sudden and severe growth defects as expression was reduced. Approximately one third of the genes did show a corresponding increase in variation as growth rate declined over at least part of the range of gene expression levels. However, many genes (e.g., POL5, LCB2, SDA1, SEC17) showed no change in variability even as growth rates approached zero, and most genes showed some changes in variation while growth rate was constant. In the case of RRN3, phenotypic potential actually approached wild-type levels only when growth was severely reduced (Fig7, Supplementary Figs S6 and S7). While the global trend suggests that a moderate degree of variation can typically be revealed before fitness is necessarily reduced, it is clear that the expression dynamics or specific functions of genes are important in determining how their roles in phenotypic stabilization and growth are related.

## Discussion

We have conducted a large-scale assessment of cell-morphology phenotypes in a set of hypomorphic and repressible alleles of 873 essential genes in yeast. Our data comprise dozens of independent morphological measurements for more than 3 million cells. Most of these genes are absent from large-scale, genome-wide screens due to reliance on the deletion library. Thus, this work represents an important step in providing standardized measures of these genes’ effects on morphology.

Our primary motivation for this study was to understand the genetic control of phenotypic robustness. We have identified at least 175 essential genes that suppress the effects of stochastic or microenvironmental variation on phenotypic variation. The proportion of phenotypic stabilizers among essential genes is therefore substantially higher than that among nonessential genes, as had been predicted (Levy & Siegal, 2008). The high proportion also supports the idea that a large number of genes in yeast promote robustness (Levy & Siegal, 2008).

The nonessential genes previously identified as stabilizers of morphological variation shared many characteristics with essential genes, including strong over-representation of GO terms for core cellular processes and highly connected positions within molecular networks. However, we were unable to identify any relationship between these measures and phenotypic potential within the 873 essential genes that we assayed. It may be that these correlations were simply missed in this study due to the high baseline levels of GO-term enrichment and protein–protein interaction network connectivity already present in the essential genes. Since some of the DAmP strains do not reduce gene expression enough to have phenotypic effects, it is also possible that a high level of false-negative results obscured correlations. Analysis of a subset of essential genes using Tet-repressible alleles suggests that this is indeed the case and that nearly all essential genes play at least a limited role in maintaining phenotypic robustness.

Another important difference between our study and previous investigations of deletion mutants is that each DAmP allele has an unknown degree of variation in expression level. It could be argued that the phenotypic variation we observed is simply a function of variable expression between cells. Although such effects cannot be ruled out entirely, we do not believe they can explain our results. Variation in protein content between cells can be well modeled with only two parameters describing transcriptional burst size and burst frequency (Cai *et al*, 2006). Factors that have been shown to regulate the kinetics of transcriptional bursting and gene expression noise include the presence of a TATA-box, nucleosome occupancy, chromatin modifications, pausing of the transcriptional apparatus, and the location of genes within the nucleus (Boeger *et al*, 2008; Choi & Kim, 2009; McCullagh *et al*, 2010; Ribeiro *et al*, 2010; Miller-Jensen *et al*, 2011). Since the DAmP alleles possess the native promoter sequences, it is unlikely that any of these processes would be perturbed. Furthermore, DAmP multiple copy arrays have been used specifically as a method to decouple the affects of gene expression noise from mean expression level (Vardi *et al*, 2013).

Our results lend support to the idea that phenotypic robustness of cellular morphology is a general property of a genotype rather than trait specific. A given strain's variability in nearly any morphological phenotype was correlated with its variability in most other phenotypes. This was true despite the fact that mean values for these same traits were not correlated (Supplementary Fig S5). Given that all of the strains we assayed are isogenic—or at least nearly so—aside from their unique single-gene disruptions, we cannot say for sure that this concept is broadly applicable to populations that contain substantial amounts of genetic diversity. A recent analysis that is highly complementary to our own may shed some light on this question. Yvert *et al* (2013) used a nearly identical morphological phenotyping method to analyze within- and between-genotype variability for a collection of wild yeast isolates representing diverse genetic backgrounds. It seems more common in their dataset than in ours that a given strain will show relatively large amounts of variance within specific sets of phenotypes but not others. However, they do identify some genotypes that are globally more variable than others. These genotypes do not cluster into a single *S. cerevisiae* lineage or habitat of origin (Yvert *et al*, 2013), suggesting that several evolutionary transitions to high global variance have occurred under different ecological pressures and that small numbers of mutations may modulate general morphological robustness in nature.

The relative evolutionary costs and benefits of robustness versus heterogeneity depend on the selective regime and, in particular, on the predictability of the environment (Levy and Siegal, 2012). The existence of differences in morphological variation between natural isolates suggests that evolution might tune robustness (Yvert *et al*, 2013). Further study will be required to determine whether these differences are indeed adaptive. Genetic mapping of the differences between wild strains could also test whether genes with high phenotypic potential are the ones harboring alleles that confer different levels of robustness, or instead whether unknown constraints prevent genes with high phenotypic potential from varying in nature. In the same vein, it is of interest to better understand how differences in morphological phenotype distributions relate to differences in growth-rate distributions. Wild isolates do differ in their growth-rate variances (Ziv *et al*, 2013). The ecological relevance of these differences is unknown as yet, but growth-rate heterogeneity has been shown in the laboratory to be plausibly adaptive by enabling a population to hedge its bets against acute environmental changes (Levy *et al*, 2012).

The study of natural genetic variation also raises important questions about the relationship between nongenetic and genetic sources of phenotypic variation. For example, is a genetic background that is prone to greater variation among isogenic cells also prone to greater average effects of new mutations? It is fair to say that the consensus prediction for many years has been that the answer to this particular question would be yes (Meiklejohn & Hartl, 2002; Masel & Siegal, 2009). Only recently has the prediction been directly tested and, in that case, the answer was no (Richardson *et al*, 2013). Similar tests of other phenotypic stabilizers, including our newly discovered essential ones, will need to be done before the connection between stochastic/microenvironmental and mutational robustness can be completely rejected.

Whether they are mechanistically connected or not, modulators of the levels of nongenetic variation and of genetic variation are potentially major drivers of adaptation. As stated above, increased nongenetic variation could constitute a bet-hedging mechanism. Seen in a slightly different way, it could enable a kind of phenotypic exploration that permits initial survival under extremely challenging conditions, prior to genetic change (Frank & Rosner, 2012). For genetic variation, a phenomenon known as capacitance could accelerate evolutionary change. A capacitor modulates the effects of genetic variation so that it accumulates neutrally then is revealed at potentially opportune times (Rutherford & Lindquist, 1998; Sangster *et al*, 2004).

One criticism that has been raised against the adaptive value of heterogeneity in natural populations is that highly variable genotypes may be unlikely to exist for long enough to find a specific environmental context for which they would be well suited. The underlying idea behind this view is that the function of a cellular network is critically dependent upon the sufficient function of each individual network component. Allowing greater variation in multiple components means that the net effect will be deleterious. Thus, cells, like families, would be subject to the Anna Karenina principle where all happy cells are alike; each unhappy cell is unhappy in its own way (Tolstoy, 1877). One argument in favor of this model is that population growth rates, often considered proxies for fitness in yeast, were found to be negatively associated with phenotypic potential in the previously published dataset of the nonessential-gene deletion collection (Levy & Siegal, 2008; Wang *et al*, 2011).

Our results support a more nuanced view of phenotypic heterogeneity, particularly in the case of nongenetic variation. We have shown that increased morphological variation does not necessarily imply decreased growth. Indeed, for the DAmP alleles as a whole, growth rate does not strongly predict phenotypic potential and, to the extent that it does, the relationship runs opposite to expectation: higher phenotypic potential correlates with higher growth rate. A more detailed analysis of individual genes using the Tet system provides more clarity. While many strains did show both increased morphological variation and reduced growth rate as expression was reduced, this pattern was not clear in most cases and was sometimes reversed. There may be a limit at which extreme levels of variability ultimately lead to growth defects, but also a range in which variation is unrelated to growth. Therefore, mutations that increase variability are not necessarily at any special disadvantage relative to those that maintain low variability. The different patterns observed in the DAmP and Tet systems suggest that subtleties in gene expression dynamics may be a critical piece of this puzzle. One likely factor is the role of noise in gene expression, which must play a role at some level in variation between isogenic cells.

Taken together, our findings indicate that a large number of nonessential genes (Levy & Siegal, 2008) and essential genes (this study) can function as phenotypic stabilizers. These results suggest that the mutational target for modulation of phenotypic stability is large. It is unclear whether severe mutations in nonessential gene or subtle modifications to essential genes are the more likely scenario for the release of phenotypic variation. Regardless, phenotypic stability has the potential to be a highly evolvable trait. Still, much work remains to understand the forces that shape levels of nongenetic heterogeneity and to identify cases in nature when increased heterogeneity has been adaptive.

## Materials and Methods

### Yeast strains

The DAmP allele haploid collection (BY4741-derived MATa his3Δ1 leu2Δ0 ura3Δ0) was purchased from Open Biosystems. Of the 879 strains listed in the collection, we were able to successfully culture all but 6 strains (CDC47, MCM6, RPS31, YLR230W, RPB11, and HSP10). The Yeast Tet-promoter Hughes Collection (BY4741-derived MATa his3Δ1 leu2Δ0 met15Δ0) was purchased from Open Biosystems.

### Yeast growth and handling for morphological analysis

Unless otherwise specified, all yeast strains were grown in liquid YPD media at 30°C in a shaking incubator. All strains were grown in 96-well plates containing 200 μl/well YPD + 200 μg/ml G418. Cells in 96-well plates were harvested by centrifugation at 172 × *g* for 2 min.

### Preparation of cells for imaging

DAmP strains were diluted 1:100 from stock plates into new plates and grown for 48 h. These saturated cultures were then diluted 1:200 into new plates, and cell density was measured for the four strains with the highest reported growth rates every 2 h. After 8 h of growth (or earlier if any one of the fastest growing strains exceeded a density of 5 × 10^6^ cells/ml), cells were harvested and fixed in PBS containing 4% paraformaldehyde (100 μl/well) for 1 h at room temperature. Cells were washed twice with PBS (200 μl/well) and then stained with a solution of 20 μg/ml FITC-conjugated concanavalin A (MP Biomedicals, 75 μl/well) for 1 h at room temperature in the dark. Cells were washed twice with PBS, resuspended in PBS (typically 150 μl/well), and stored at 4°C for up to 1 week. Cells were gently sonicated using a Misonix ultrasonic liquid processor with a 96-probe tip set at amplitude 10 with 10 1-s pulses immediately before mounting.

For each plate, 5 ml of mounting media was prepared by mixing 2.5 ml VectaShield, 2 ml PBS, and 500 μl DAPI (1 μg/ml). 50 μl of mounting media was added to each well of a glass-bottom 96-well plate (Matrical). 50 μl freshly sonicated cell suspension was then added to each well and mixed thoroughly. These plates were centrifuged at 689 × *g* for 2 min prior to imaging.

### Image collection and analysis

Images were collected using a Nikon TE2000 microscope outfitted with a perfect focus system, a Prior automated stage, a Nikon Intensilight light source, and a QI Click 2.4-megapixel camera. Typically, 65–100 fields/well were captured in two channels configured for FITC and DAPI, respectively. Unlike the original protocol (Ohya *et al*, 2005), ours did not include a third channel with a filamentous-actin stain (phalloidin), as we had determined that actin-based CalMorph phenotypes contributed little to analysis of morphological variation (Richardson *et al*, 2013). Raw tiff images were processed using custom software to produce 696 × 520 8-bit jpeg images. These images were then analyzed using the CalMorph software package (Ohya *et al*, 2005).

### Data quality control

All CalMorph data were processed in the R programming environment. Any genotypes that did not contain at least 200 images each of G1–S, S–G2, and M-phase cells were removed from further analysis. CalMorph phenotypes with codes C127, D160, D164, D171, D188, and D189 were eliminated from the dataset due to annotation issues or because they measured distances on the order of one or two pixels. Any phenotypes that contained more than 3% missing values were removed from analysis. Any cells with missing values for any remaining phenotypes were removed from the dataset. Discontinuous or multimodal phenotypes were identified by the dip test. Any phenotypes with a dip statistic greater than 0.05 were removed from further analysis. Cells with any phenotypes that were more than 10 standard deviations from the mean were removed from the dataset because manual inspection suggested that such outliers were always artifactual. Linear modeling was performed to determine the effect due to replicate plates. Any phenotypes for which 20% or more of the total variance was explained by replicate effects were removed from the dataset. A full list of the CalMorph phenotypes used in this study is in Supplementary Table S1.

### Data analysis

All data analysis was performed in the R programming environment. The complete dataset was divided into three matrices based on the major CalMorph phenotypic groups (A, A1B, and C; corresponding to G1–S, S–G2, and M–G1, respectively). Each phenotype (column) was Box–Cox-transformed to produce an approximately normal distribution. Each value was subtracted from its column mean and divided by its column standard deviation so that each phenotype had a mean of 0 and a standard deviation of 1. Dimensional reduction was performed by principal component analysis such that >90% of the total variance was retained with a minimal number of components. Loess regression was performed on the variances within each genotype with respect to their mean values for each remaining principal component. The residuals were taken as the mean-corrected variances, and these were normalized such that the total variance of the individual, mean-corrected variances, across all genotypes, within each component, was equal to 1. The phenotypic potential of a genotype was then calculated by averaging the variances across the 50% of the principal components that had the largest variances for that genotype. The false discovery rate was estimated by performing 100 random permutations of the residual variances within each principal component and determining the fraction of random permutations with a value at least as large as a given phenotypic potential score.

### Growth-rate measurement

All of our growth-rate data were obtained using time-lapse microcolony microscopy as described previously (Levy *et al*, 2012). Similar methods were used to measure the percentage of dead cells. In this case, the growth medium was supplemented with 1 μg/ml propidium iodide. Cells were imaged under bright field and fluorescence. At the first time point, each cell was called as either living or dead based on the fluorescence intensity. It was also verified that no cells that were called as dead produced any progeny.

### Data availability

Summaries of several datasets associated with this study are available as Supplementary Datasets S1, S2, S3, S4, S5 and S6. All of the images and raw morphological data associated with this study are publicly available through the Dryad data repository (doi:10.5061/dryad.ft7dj). Any additional data is available upon request by contacting Mark Siegal (mark.siegal@nyu.edu).

## Supporting Information

Supplementary Dataset S6

Analysis Scripts

Review Process File
