# Supplementary figures and images for: Essential gene disruptions reveal complex relationships between phenotypic robustness, pleiotropy, and fitness
Source: Mol Syst Biol. 2015 Jan 21;11(1):773. doi: 10.15252/msb.20145264 (PMC4332149; doi:10.15252/msb.20145264)

## Rank Correlations of Phenotypic Potential Scores

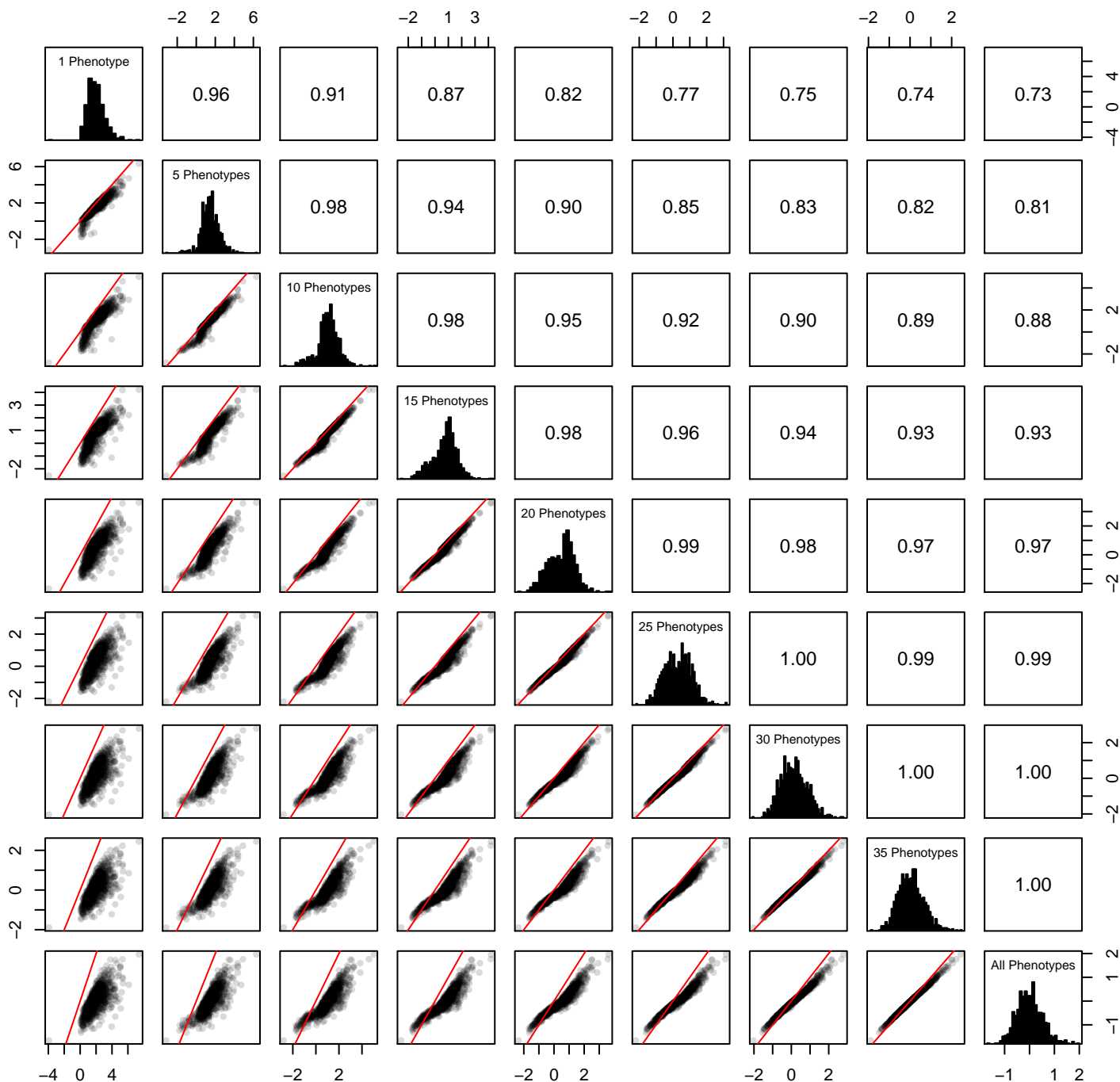

Supplement: Supplementary file 1 [file msb0011-0773-sd1.pdf]

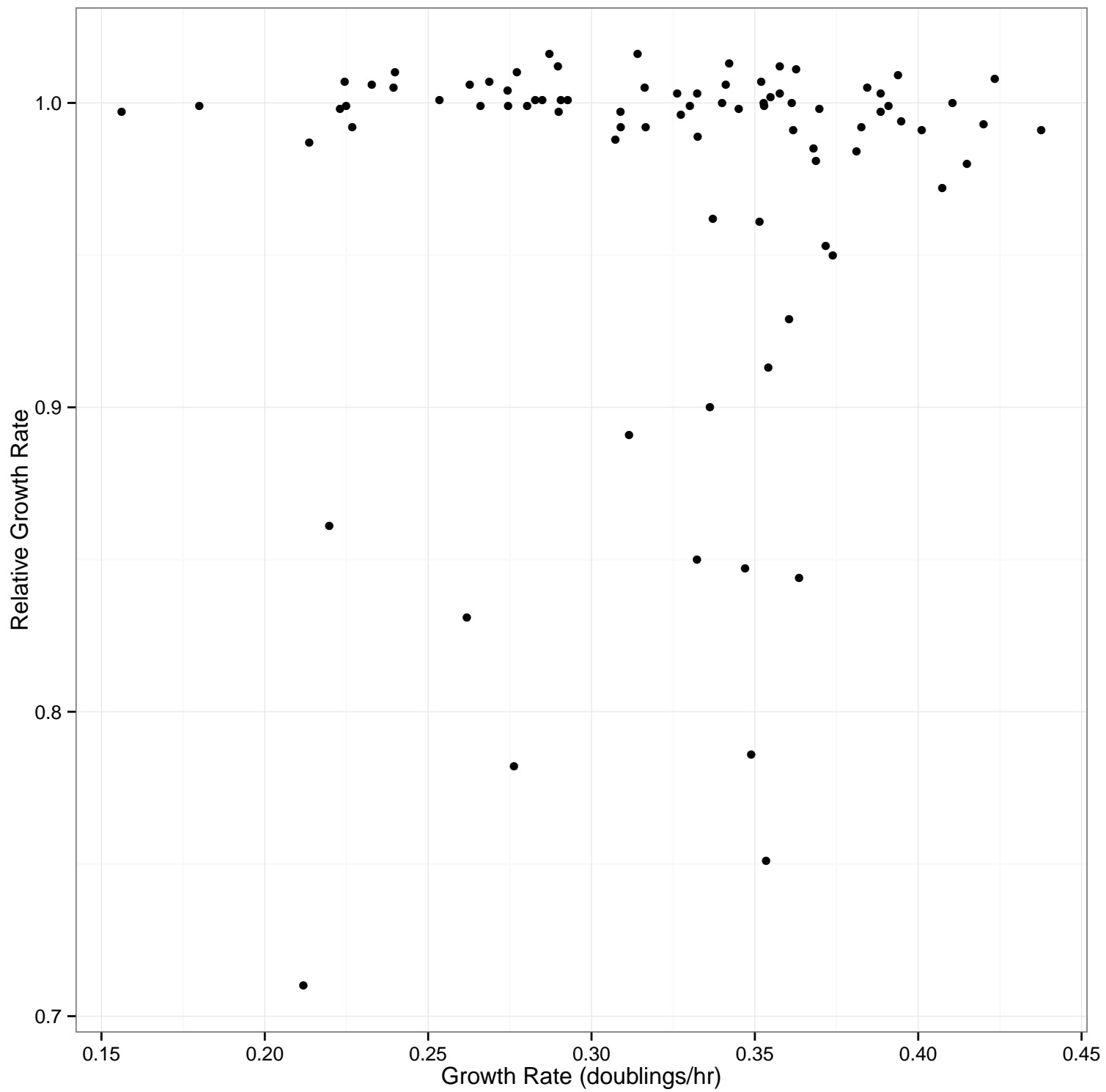

Supplement: Supplementary file 2 [file msb0011-0773-sd2.pdf]

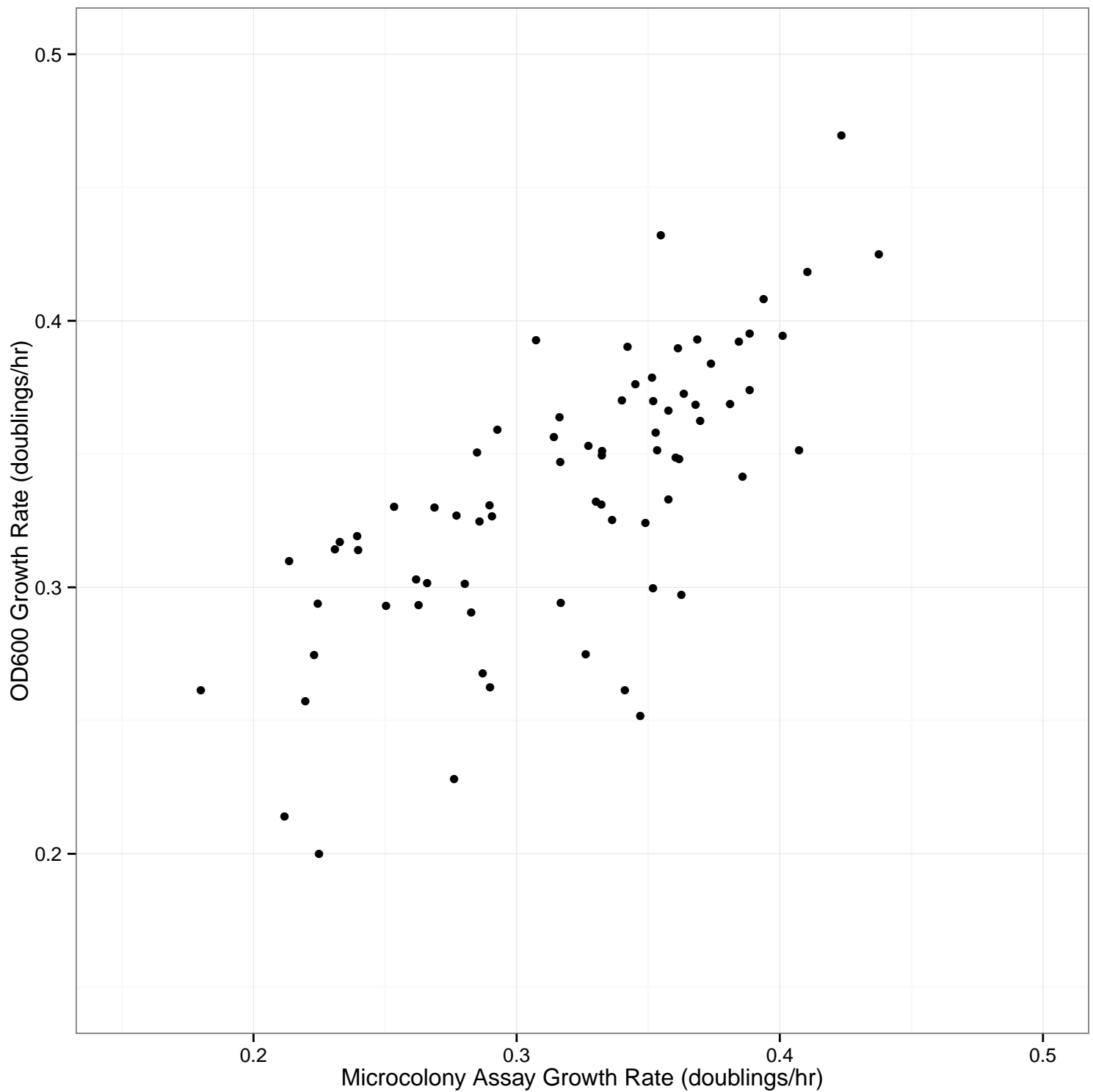

Supplement: Supplementary file 3 [file msb0011-0773-sd3.pdf]

Phenotypic Potential vs Genetic Interactions

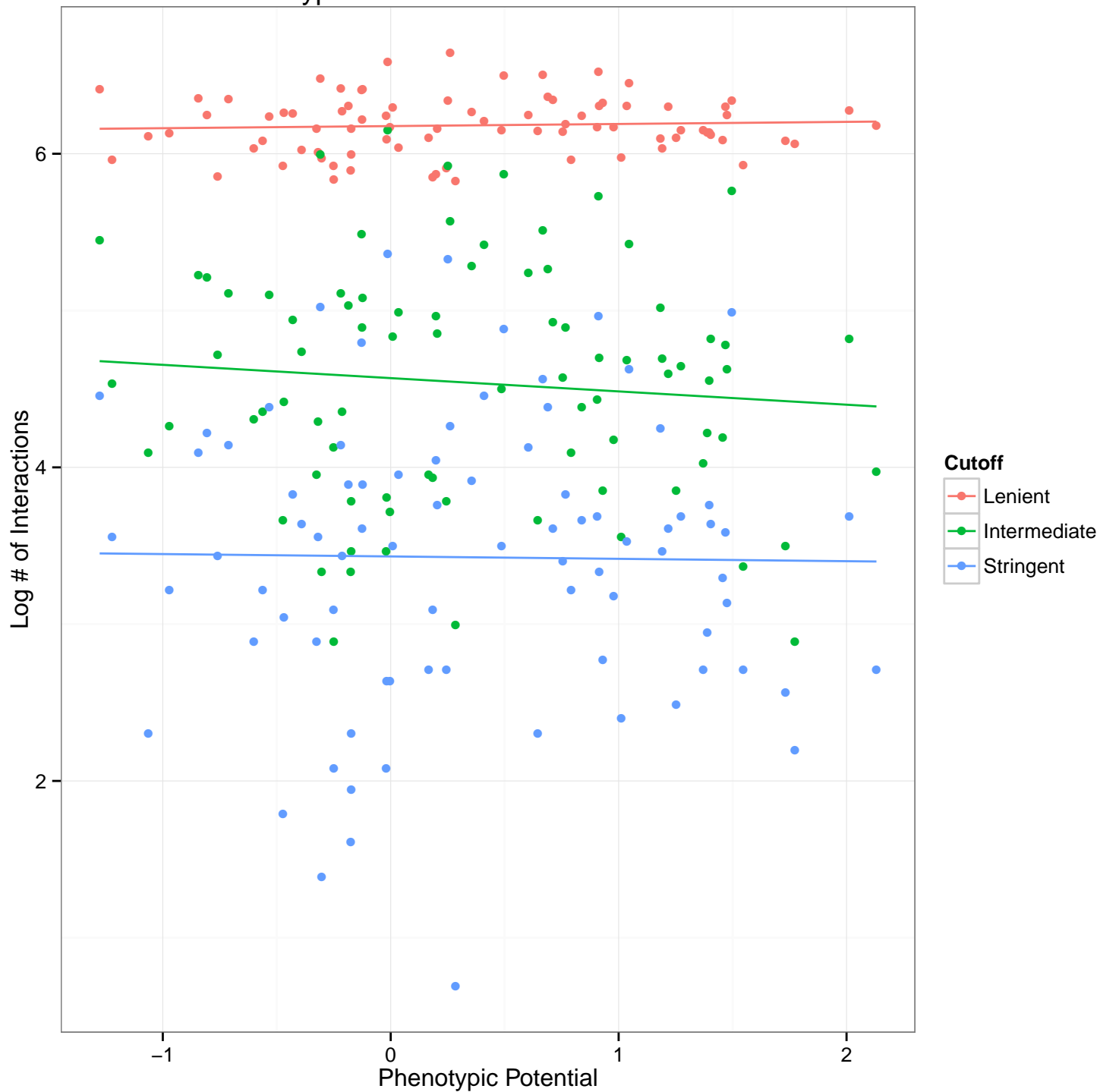

Supplement: Supplementary file 4 [file msb0011-0773-sd4.pdf]

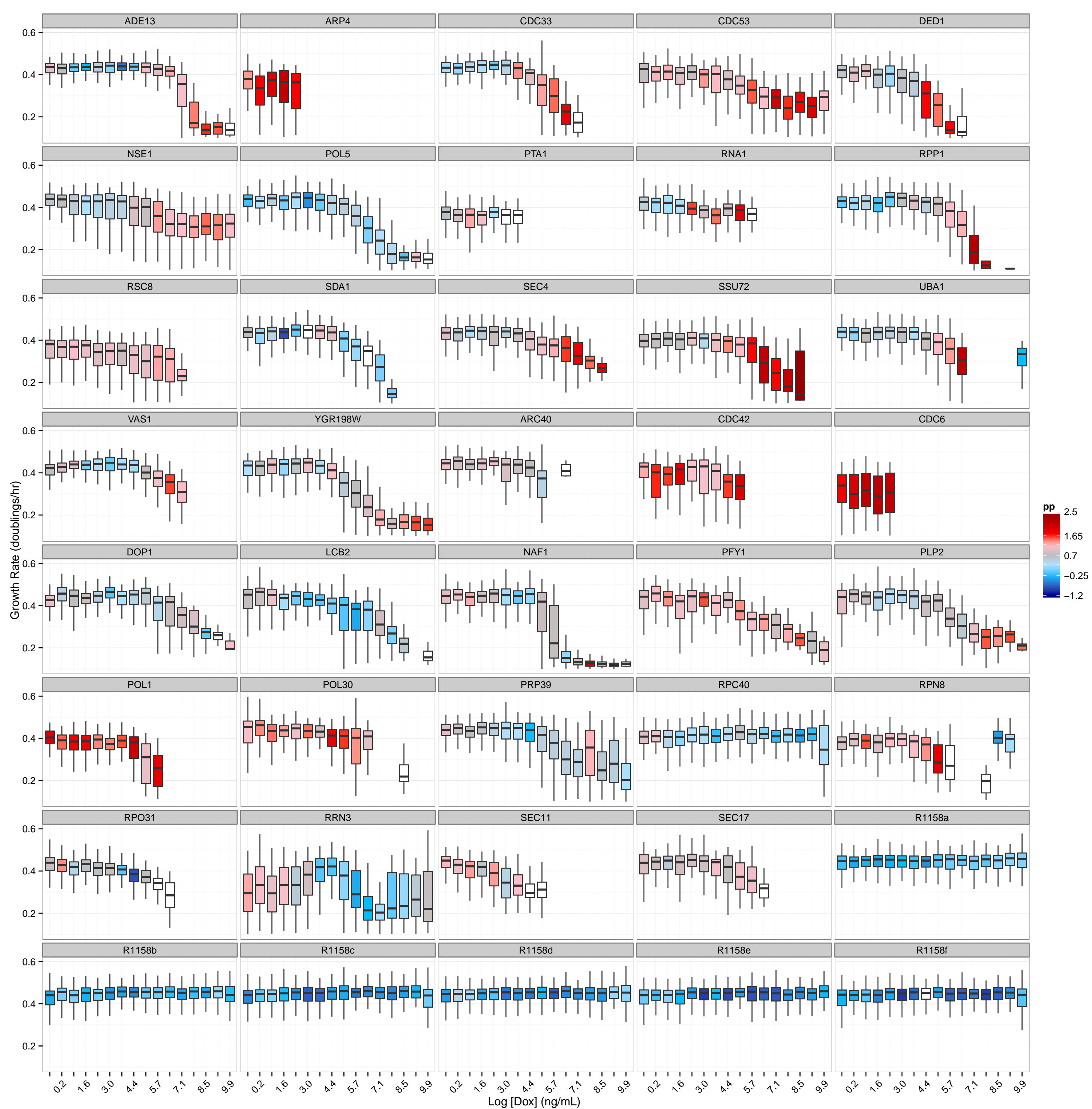

Supplement: Supplementary file 6 [file msb0011-0773-sd6.pdf]

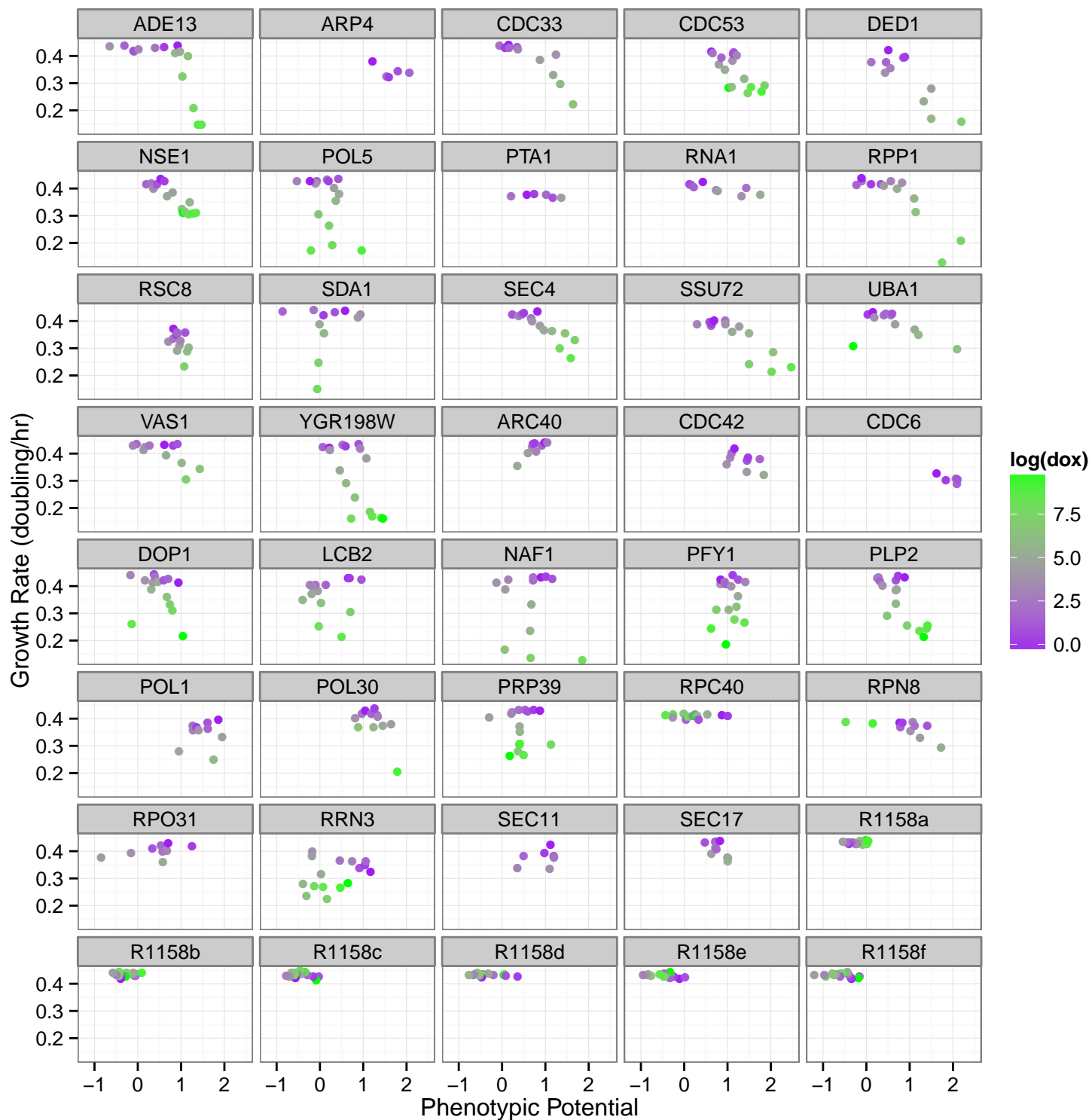

Supplement: Supplementary file 7 [file msb0011-0773-sd7.pdf]

# PCA a

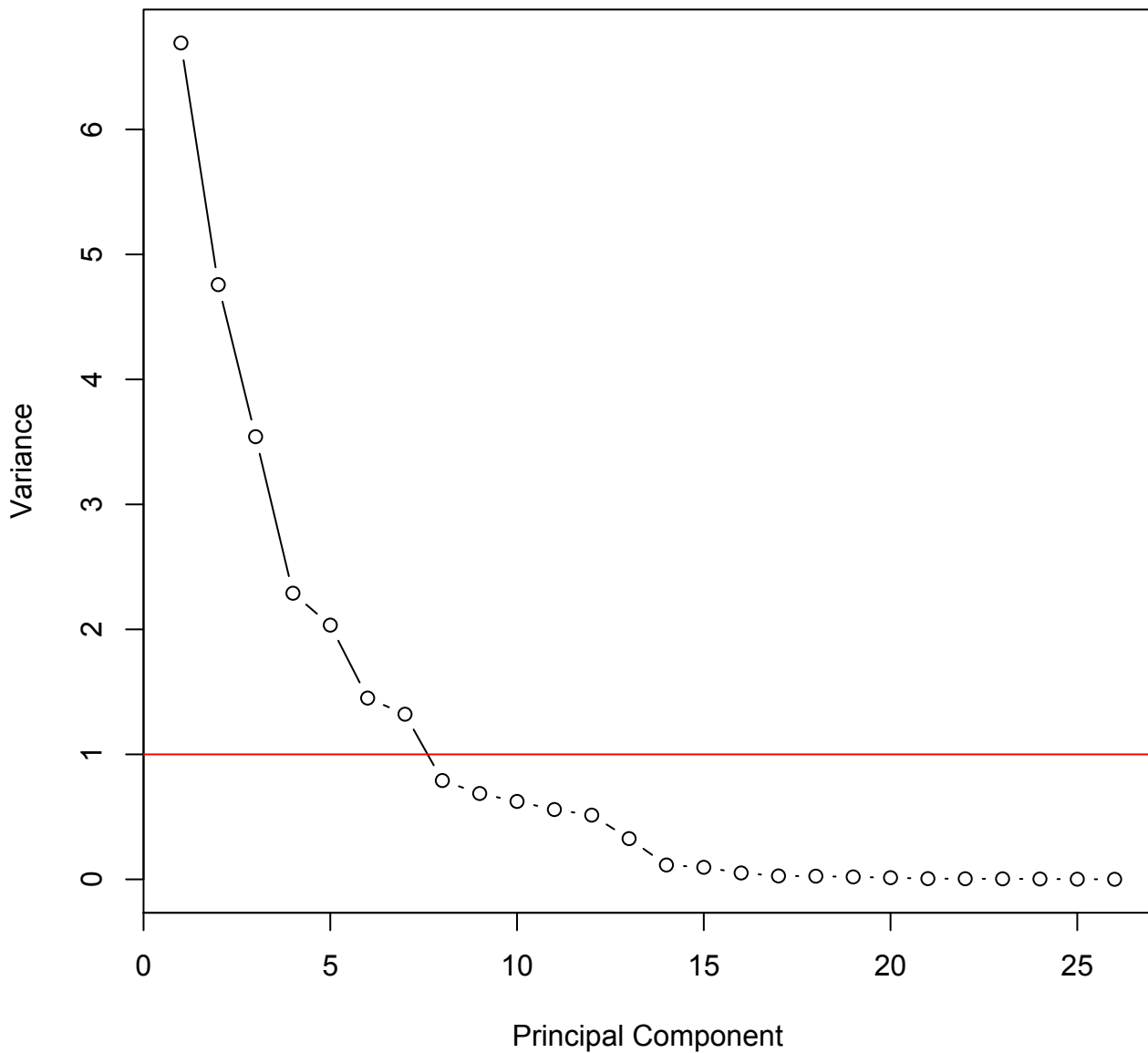

Supplement: Supplementary file 8 [file msb0011-0773-sd8.pdf]

# PCA b

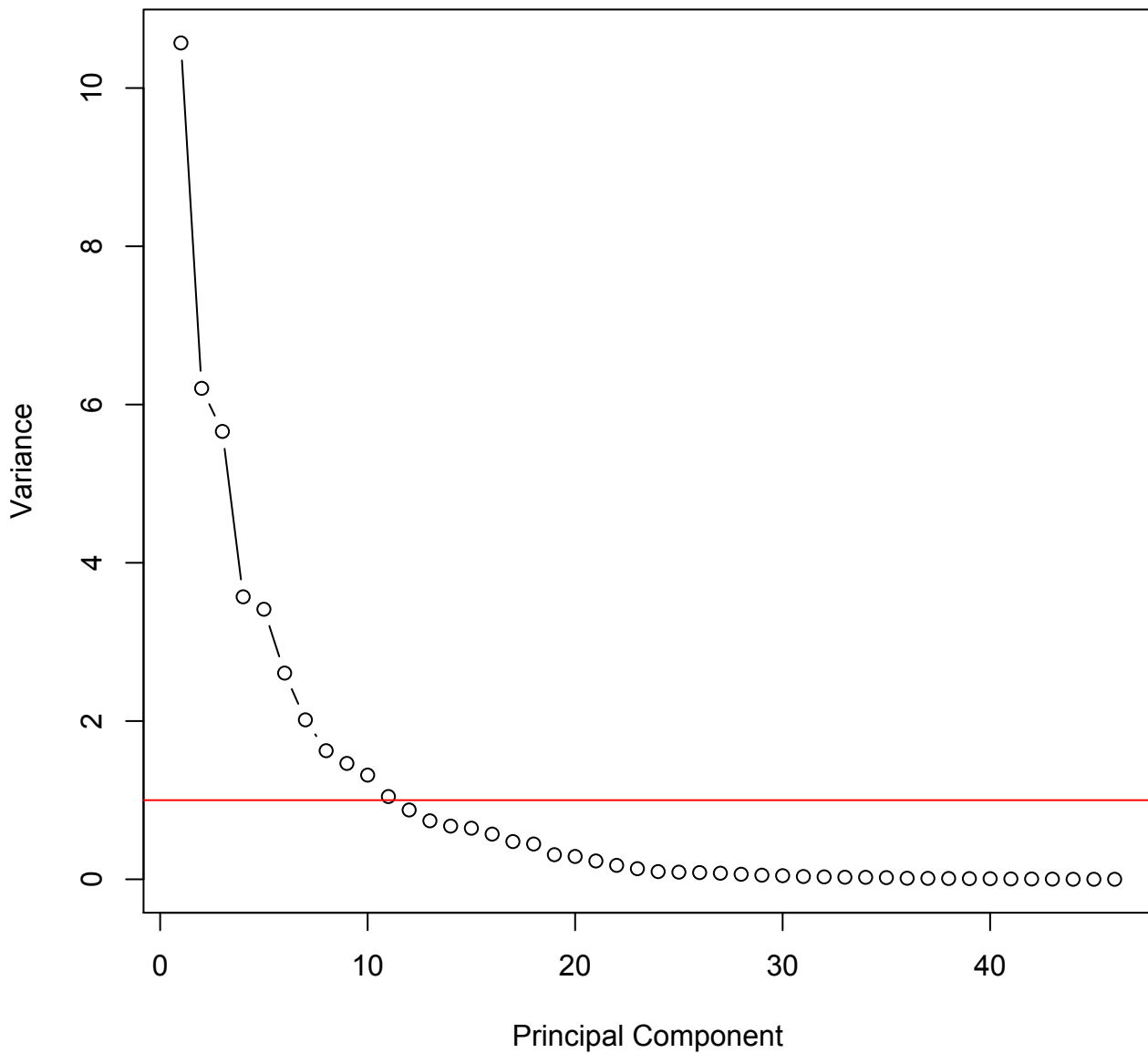

Supplement: Supplementary file 9 [file msb0011-0773-sd9.pdf]

# PCA c

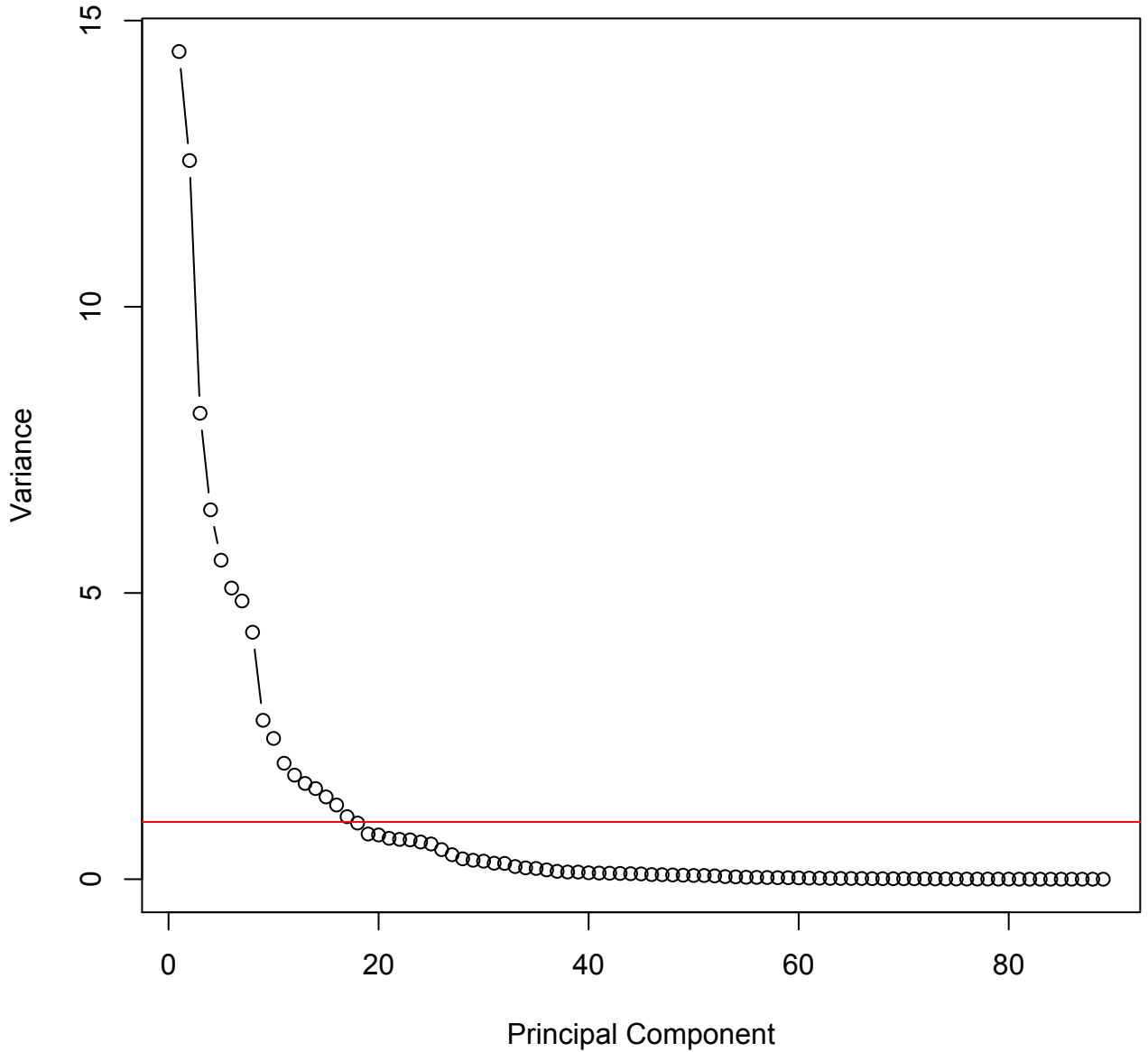

Supplement: Supplementary file 10 [file msb0011-0773-sd10.pdf]

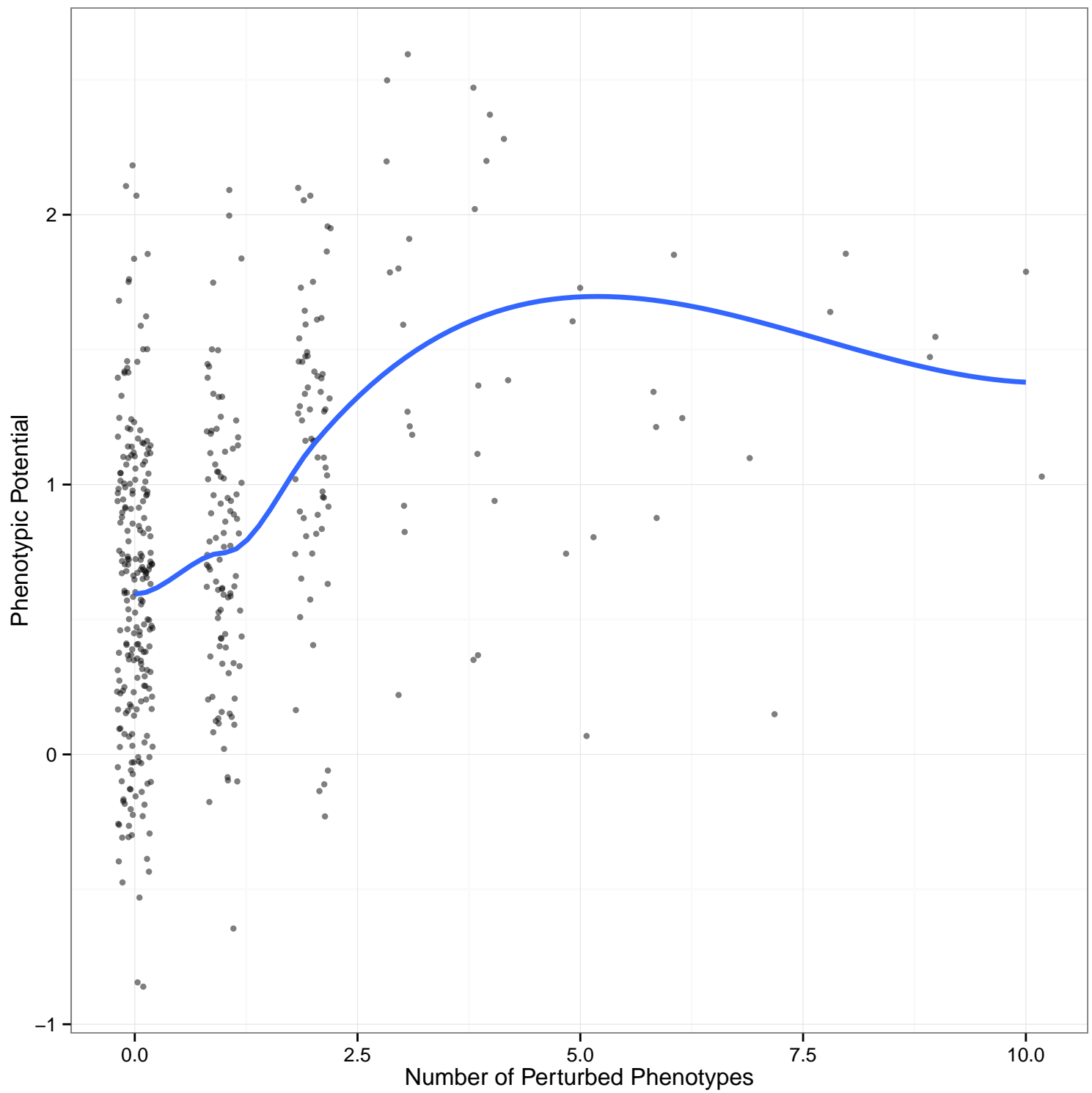

Supplement: Supplementary file 11 [file msb0011-0773-sd11.pdf]
