# Supplementary material for: Essential gene disruptions reveal complex relationships between phenotypic robustness, pleiotropy, and fitness
Source: Mol Syst Biol. 2015 Jan 21;11(1):773. doi: 10.15252/msb.20145264 (PMC4332149; doi:10.15252/msb.20145264)
Supplement: Supplementary file 5 [file msb0011-0773-sd5.pdf]

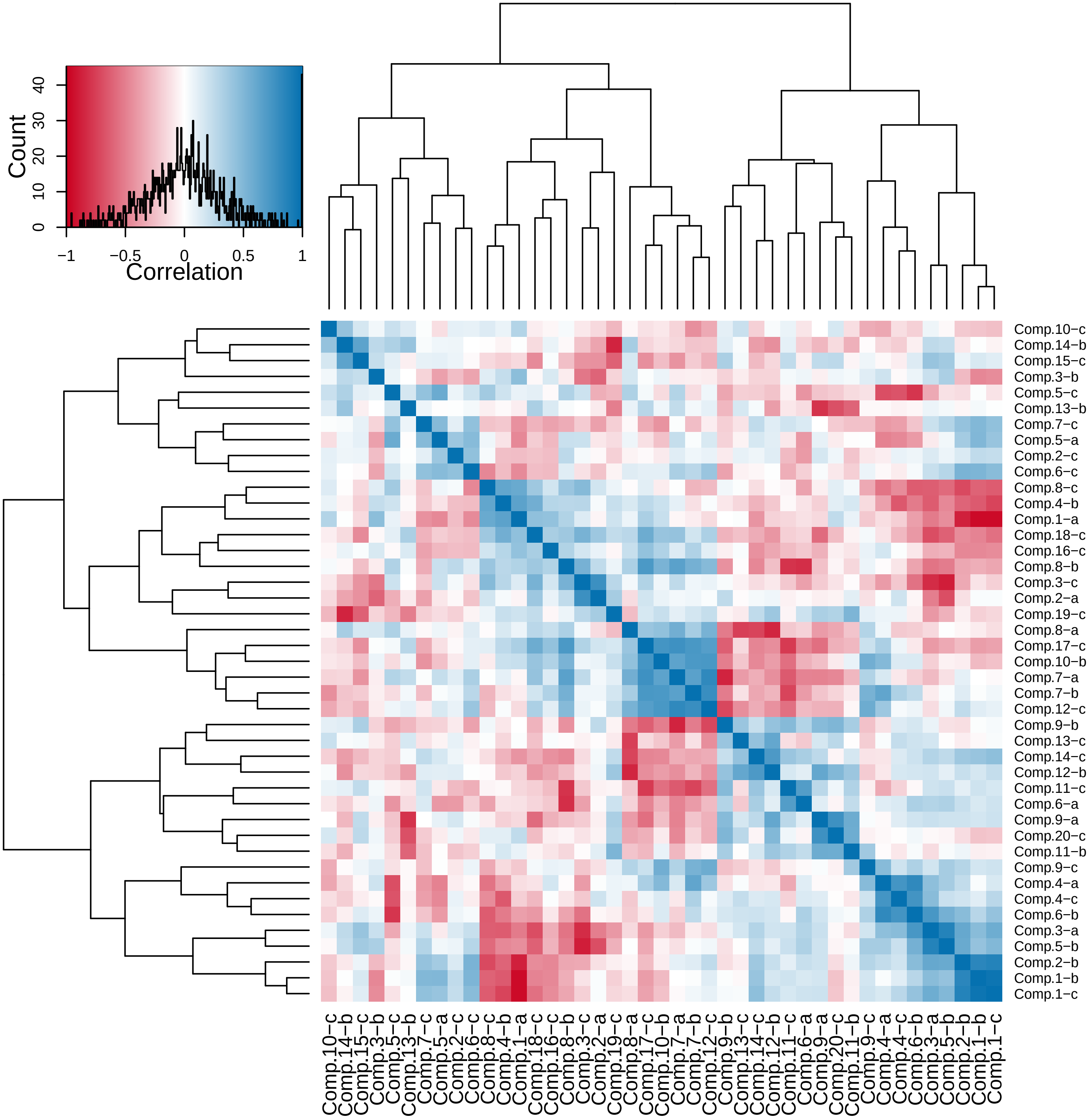
Count
0
10
20
30
40
−1
−0.5
0
0.5
1
Correlation
Comp.10−c
Comp.14−b
Comp.15−c
Comp.3−b
Comp.5−c
Comp.13−b
Comp.7−c
Comp.5−a
Comp.2−c
Comp.6−c
Comp.8−c
Comp.4−b
Comp.1−a
Comp.18−c
Comp.16−c
Comp.8−b
Comp.3−c
Comp.2−a
Comp.19−c
Comp.8−a
Comp.17−c
Comp.10−b
Comp.7−a
Comp.7−b
Comp.12−c
Comp.9−b
Comp.13−c
Comp.14−c
Comp.12−b
Comp.11−c
Comp.6−a
Comp.9−a
Comp.20−c
Comp.11−b
Comp.9−c
Comp.4−a
Comp.4−c
Comp.6−b
Comp.3−a
Comp.5−b
Comp.2−b
Comp.1−b
Comp.1−c
