# Supplementary material for: Essential gene disruptions reveal complex relationships between phenotypic robustness, pleiotropy, and fitness
Source: Mol Syst Biol. 2015 Jan 21;11(1):773. doi: 10.15252/msb.20145264 (PMC4332149; doi:10.15252/msb.20145264)
Supplement: Supplementary file 12 [file msb0011-0773-sd12.docx]

**Supplemental Figure S1**

Phenotypic potential is robust to the number of phenotypes included. The diagonal panels show the distributions of phenotypic potentials based on the displayed number of phenotypes. The lower-left panels show scatter plots of genotypes comparing the phenotypic potential scores between each pair of phenotype thresholds with red lines indicating the line y=x. The upper-right panels show the Spearman rank correlation coefficients for each comparison.

**Supplemental Figure S2**

Comparison of micro-colony based growth rate measurements and the published relative growth rates from competition assay (Breslow et al. 2010) for the DAmP alleles with the 48 highest and 48 lowest phenotypic potentials.

**Supplemental Figure S3**

Comparison of micro-colony based growth rate measurements and bulk population growth rates based on OD measurements from a plate reader.

**Supplemental Figure S4**

Relationship between phenotypic potential and number of genetic interactions. For a subset of DAmP strains, phenotypic potential is plotted against the number of genetic interactions (Costanzo et al, 2010). Three stringency levels are shown ranging from the most lenient (red) to the most conservative (blue).

**Supplemental Figure S5**

Pairwise correlations between phenotype means. Each strain in the DAmP collection was assigned a mean for each of the top 41 principal components. The distribution of correlation coefficient values between the mean values of each pair of phenotypes, across all strains, is shown in the upper left panel. The intensity of the blue shading in the heat map indicates the degree of positive correlation while red shading indicates the degree of negative correlation. The dendrograms were determined by hierarchical clustering.

**Supplemental Figure S6**

Dynamics of changes in growth rate and phenotypic potential for individual genes as a function of expression level. In each case, the levels of doxycycline range from zero on the far left to 20 μg/mL on the far right. Box plots show the distributions of growth rates observed with color shading to indicate the observed phenotypic potential (blue=low variation, red=high variation, white boxes indicate too few cells to calculate a phenotypic potential, and the absence of a box indicates fewer than 10 colonies observed).

**Supplemental Figure S7**

Dynamics of changes in growth rate and phenotypic potential for individual genes as a function of expression level. In each case, growth rate is plotted against phenotypic potential. The points are shaded to indicate the concentration of doxycycline ranging from zero (purple) to 20 μg/mL (green).

**Supplemental Figure S8**

Results of principal component analysis for unbudded cells. The principal components are plotted in order along the x-axis with the associated variance on the y-axis. The redline indicates of the variance of the normalized CalMorph parameters. Enough components were kept to explain at least 90% of the total variance.

**Supplemental Figure S9**

Results of principal component analysis for small-budded cells. The principal components are plotted in order along the x-axis with the associated variance on the y-axis. The redline indicates of the variance of the normalized CalMorph parameters. Enough components were kept to explain at least 90% of the total variance.

**Supplemental Figure S10**

Results of principal component analysis for large-budded cells. The principal components are plotted in order along the x-axis with the associated variance on the y-axis. The redline indicates of the variance of the normalized CalMorph parameters. Enough components were kept to explain at least 90% of the total variance.

**Supplemental Figure S11**

Relationship between pleiotropy and phenotypic potential. For each combination of Tet strain and concentration of doxycycline, the number of phenotypes that differ from the wild type reference by at least one standard deviation are plotted on the x-axis. A small amount of noise was added to prevent over plotting. Phenotypic potential scores are plotted along the y-axis. The blue line indicates the result of a loess regression.

**Supplemental Table S1**

List of curated CalMorph morphological parameters that were used in this study.
