# Supplementary material for: Essential gene disruptions reveal complex relationships between phenotypic robustness, pleiotropy, and fitness
Source: Mol Syst Biol. 2015 Jan 21;11(1):773. doi: 10.15252/msb.20145264 (PMC4332149; doi:10.15252/msb.20145264)
Supplement: Supplementary file 21 [file msb0011-0773-sd21.pdf]

Essential gene disruptions reveal complex relationships between phenotypic robustness, pleiotropy, and fitness

Christopher R. Bauer, Shuang Li, Mark L. Siegal

Corresponding author: Christopher R. Bauer, NYU

Review timeline:

Submission date:	10 March 2014
Editorial Decision:	09 May 2014
Revision received:	25 August 2014
Editorial Decision:	16 October 2014
Revision received:	17 November 2014
Accepted:	30 November 2014

Editor: Thomas Lemberger

Transaction Report:

(Note: With the exception of the correction of typographical or spelling errors that could be a source of ambiguity, letters and reports are not edited. The original formatting of letters and referee reports may not be reflected in this compilation.)

1st Editorial Decision 09 May 2014

Thank you again for submitting your work to Molecular Systems Biology. We have now heard back from the three referees who agreed to evaluate your manuscript. As you will see from the reports below, the referees find the topic of your study of potential interest. They raise, however, substantial concerns on your work, which, I am afraid to say, preclude its publication in its present form.

The reviewers recognize the quality of the dataset and its potential value as a resource to the community. They raise however serious issues with regard to the conclusiveness of the analysis and the interpretation of the data. Without repeating all the issues noted by the reviewers, the major points that need to be convincingly addressed refer to the following:

- additional alternative ways to quantify/analyze pleiotropy (eg alternative metrics that do not combine both number and magnitude of phenotypic alterations, anaysis on complementary data types) are required to confirm the robustness of the conclusions.

- the conclusion with regard the effects on fitness should be more systematically validated and confirmed rigorously with alternative growth measurement methods.

Reviewer #1:

The authors show that the extent of cell morphological variability in yeast strains where essential genes are reduced in function using UTR insertions (DAmP alleles) does not correlate with the growth defect in each strain. In addition, using principle components analysis, they show that morphological variation tends to be correlated across 'phenotypes' i.e. that strains with stronger

© European Molecular Biology Organization

morphological defects tend to have multiple morphological defects. The basic dataset is interesting, but I think more could be got from it to raise the analysis to the level of MSB. In particular, I am not convinced that one of the main conclusions (phenotypic potential correlates (weakly) with phenotypic distance) is not trivial, given how these measures are calculated. Moreover the negative results (growth rate vs potential) would be firmer if more independent datasets were analyzed.

Comments

Pleiotropy. I think the use of pleiotropy in the title is somewhat misleading, given that here pleiotropy is defined from the morphological data. i.e. my interpretation is that there are typically pleiotropic morphological defects when morphology is affected, not that these 'correlate' with pleiotropy. There are many other ways to quantify pleiotropy from different data types e.g. from genetic interaction profiles (see e.g. Bellay et al. GR 2011) or from functional annotations or from physical interactions. The authors could investigate how these relate to morphological variability. However, I think the more straightforward conclusion is that cells with more severely affected morphological phenotypes tend to have more affected morphological phenotypes.

Sensitivity to measures used. The phenotypic potential measure is designed to capture the variability and magnitude of defects across many orthogonal morphological phenotypes. Is it really surprising therefore that strains in which phenotypes are strongly perturbed (distance measure) also have a higher potential? Especially given that this relationship is not exactly strong (see below). To quote the authors, 'This metric summarizes both the number of phenotype means that are affected by a gene's deletion as well as the magnitude of those effects, just as phenotypic potential summarizes the number and magnitude of effects on variation.' I would suggest the authors need to also present analyses that use alternative measures of both pleiotropy and potential that don't capture both number and magnitude so much: the number of traits affected is what is normally meant by pleiotropy, not how strongly each is affected.

Growth rate measurements. Growth rate datasets in yeast notoriously do not correlate well when compared across datasets/labs/techniques (see e.g. Baryshnikova et al. Nature methods 2010). How well do the fitness measures used here correlate (liquid vs colony assay etc)? In addition, microlony growth rates can underestimate population growth rates, so I think it important to use more than one dataset of bulk growth rates in the analyses before concluding a negative result. In addition, the Tet data also seems to potentially contradict the main conclusion (growth rate does seem related to potential when considered within or across the Tet strains) - this data should also be directly plotted (potential vs growth rate) and examined for each Tet strain and also across the strains.

Growth rate in yeast is very likely to be a fitness-related trait. However, morphology is not necessarily a fitness-related trait. Thus, I think the use of 'unfit' in the discussion is also misleading.

Comparison with the non-essential deletion collection data. The authors should re-analyze the deletion collection data with their new PCA-based analysis of morphological variation + a range of different datasets of growth rate. Is the lack of correlation with growth rate also upheld in the non-essential deletion collection data (after, for example, excluding deletions with no significant variation)? If not, why not?

Fig 5. The correlation between phenotypic potential and mean phenotypic distance is not strong and seems to be largely driven by a small(ish) number of data points with distance >4. How strong is the correlation for the bulk of the dataset with distance <4? If strong, presenting this bulk data as a separate plot would be appropriate.

Fig 6. dead cell censoring. Removing the dead cells, do the 'low' phenotypic potential (more dead cells) and high phenotypic potential still differ in potential? i.e. are dead cells actually less variable in morphology (?).

abstract - delete 'novel system' (it is a previously used + described mutant collection)

data availability - all of the cellular phenotype data, growth rates and rate variances analysed in this paper should be provided as data files in the supplement

Intro 'However, an alternative hypothesis...'. To me, these two models do not seem mutually exclusive alternatives.

Refs. Paaby A, Rockman M (2013) is incomplete; Lehner PlosG 2010 probably refers to PLosOne 2010 (?). Or Park/Lehner MSB 2013 (genetic hubs/capacitors have stable gene expression)?

"The flattening of the curve for distances greater than 4 is primarily due to a small number of mutants that have extremely large cell size and the fact that cell size is measured separately in each of the cell cycle categories." What's the data to support this statement?

"Pleiotropy is, by far, the strongest correlate of phenotypic potential that we have identified. " Perhaps I have missed it, but please list all other correlates that were examined, either in supplement or simply in the text. 'by far' is also overselling the result, given the relatively weak correlation.

The Anna Karenina principle - including the quotation would help the less literary reader (!)

Figure axes should be more clearly labelled and indicate the units.

Reviewer #2:

In this manuscript, Chris Bauer and colleagues analyse the consequence of reducing the activity of yeast essential genes on the variability of cellular morphology. The study follows previous work of this lab on the effect of non-essential genes on this variability. Here, Bauer et al. used a collection of 873 DAmP mutants, where expression of the target gene is reduced by destabilization of the mRNA. They processed these mutants on automated analysis of fluorescence microscopy images and extracted measures of cell shape, size and DNA distribution pattern. They found that the majority of mutants display high phenotypic potential (a metric reflecting global cell-cell variability in several unrelated phenotypes), that this phenotypic destabilization was global (as opposed to specific to a few traits), that it correlated with pleiotropy (mutants with large alteration of mean trait values also display large variability) and, importantly, that it did not correlate with cell division rate (fitness).

The work is abundant and was thoroughly done. Remarkable qualities of the study are the large dataset (triplicates per strain), careful quality control to exclude unreliable phenotypic measures, the use of PCA to define independent traits, the validation of a subset of mutations effect by using a different type of gene silencing (doxycyclin-dependent transcrptional repression), and the single-cell resolution fitness assay wich allowed to disconnect cell-cell variability in morphology from cell-cell variability in growth rate (Fig6). The conclusion that essential genes act as capacitors comfort previous expectations (because these genes tend to be network hubs). But the absence of association between phenotypic destabilization and growth rate is novel and important. It also excludes the possibility that increased phenotypic potential merely reflects a general sickness of cells that are nearly dying because of the reduced activity of an essential gene.

The manuscript can nonetheless be improved by addressing the following points:

1. How does pleiotropy (of mean phenotypic values) varies along dox concentrations of the repressible strains? A dose effect on pleiotropy could reinforce or refine the correlation observed on the DAmP strains.

2. The raw single-cell data should be made available. This is very important to allow the community to reproduce the analysis and conclusions and to allow other future investigations of the data.

3. The subsection title "Robustness can be generalized across phenotype space" is confusing. Why not speaking about pleiotropy of phenotypic variability? This will increase consistency with the subsequent section "Pleiotropy is Strongly..." which refers to pleiotropy on mean phenotypic values.

4. Sentences "Moreover, there was no general... was -0.054" should be removed. It is not surprising: sd vs. mean dependence can be positive for some phenotypes and negative for others, or even non-monotonic. Seeing a global correlation near zero across all phenotypes does not add information on the degree of sd<->mean dependence for each trait.

5. A long paragraph of the discussion repeats the conclusions of the Richardson 2013 paper. This is too long and distracting. The study here focuses on buffering against environmental (stochastic) variation: it is confusing to spend lengthy text on the buffering against genetic variation. The distinction will be clearer if the paragraph is shortened.

6. Results of the PCA defining the phenotypes should be presented (histogram of variance explained per component, dot plots of strains along PC1,PC2...)

7. The phenotypic potential values of DAmP strains (-0.77, 0.45; 0.85, 2.54, 1.99, 1.81) appear only in text. They should be printed or visualized on the figure to show the consistency between DAmP and the dox strains.

8. Methods, page32: Normalization of loess residuals. It is not clear to me if the "variance of the variances" is the variance of the residuals.

9. End of discussion: I agree that the results show that the "mutational target for modulation of phenotypic stability is large", but the authors should also discuss whether the mutational target is constrained or not: on one hand, it contains many essential genes, which do not evolve as freely as non-essential genes. On the other hand, the study shows that partial reduction of these genes' activity does not necessarily reduce fitness, at least in one environmental condition.

10. Text on page 21 should refer to Figure 7.

11. Saying that "the field of yeast genetics has strongly relied upon gene knockout" is incorrect given the contributions of mid-XXth century. The authors probably meant "genomics" and not "genetics".

12.
- page 18: shape variation these traits: "of" these traits.
- Page 24. Ruled "out" and not "our"

Reviewer #3:

In this paper, Siegal and colleagues use HTP fluorescence microscopy to image 873 haploid yeast strains harboring so-called DAmP alleles of essential genes. The images were then analyzed using CalMorph (Yoshi Ohya's software) to measure 187 "phenotypes" that were reduced (using PCA) to a set of 41 phenotypes that explained more than 90% of total variance. The authors developed a "phenotypic potential" score to identify essential genes that act as general buffers of phenotypic variation. A phenotypic potential score is produced by calculating the average standard deviation based on the top 20 (out of 41) most variable phenotypes for each DAmP strain - after applying appropriate thresholds, the authors find between 20% of genes assayed (using a random permutation method) or 52% of genes assayed that exhibit high phenotypic potential and thus act as phenotypic stabilizers. This is significantly higher than for non-essential genes, showing that essential genes are enriched for phenotypic stabilizers.
The authors then analyze their data to discover a strong positive correlation between phenotypic potential and pleiotropy, but not with fitness (using a micro-colony growth assay to measure fitness of 48 DAmPs with the greatest phenotypic potential scores and 48 DAmP alleles with the lowest phenotypic potential scores). A similar trend was not observed when comparing phenotypic potential to DAmP allele fitness measurements derived from an independent study (Breslow et al). Finally, 8 genes were selected that span a range of phenotypic potential scores and phenotypes for these genes were measured using strains carrying TET-repressible alleles of the genes - in this experiment, 6 of the genes showed the same high or low phenotypic potential seen with the DAmP allele strains, while 2 DAmP alleles with low potential exhibited much high phenotypic potential when assayed as TET alleles suggesting that DAmP alleles result in variable reduction of essential gene expression and that many essential genes with high phenotypic potential may have been missed by screening the DAmP collection.
This is an interesting study, and the analysis of the phenotypes associated with DAmP allele strains using CalMorph should be a useful resource for the community. There are some serious issues with the paper as it now stands, though, which I outline below.

[1] The following statement is misleading: "Regardless of the phenotypic potential threshold we used, essential phenotypic stabilizers are highly enriched, relative to the set of all genes, for many GO terms related to RNA processing, ribosome biogenesis, transcription, phospholipid biosynthesis, and nuclear genome organization. However, there was not any statistically significant enrichment beyond that already present within the set of essential genes." In other words, there was no statistical enrichment among the set of genes tested which is not surprising given that essential genes are, in general, enriched for these features.

[2] The authors claim that phenotypic stabilizers in their dataset tend to be pleiotropic - the strongest trend identified. I'm a little concerned that the metric used to measure pleiotropy is based on the same dataset used to measure phenotypic potential. This statement is unclear: "Moreover, there was no general underlying trend in the relationship between mean and variation within our data set to begin with". If this is true, why was loess used to begin with.

[3] I am also concerned about whether the sampling error on the mean for a given strain and phenotype is directly proportional to the variance in that phenotype for that strain. It's possible that sampling error, when averaged across a large number of phenotypes, may partially explain the observed correlation between the phenotypic distance and phenotypic potential measurements (Fig. 5). Can the authors rule this out?

[4] The authors conclude that phenotypic potential is correlated to pleiotropy but not correlated with fitness - a conclusion they state in their title. The contrast between the conclusions made in these statements is striking given that they're supported by similar statistics. For example, "A plot of phenotypic distance versus phenotypic potential reveals a strong positive relationship with Spearman's rho (R) = 0.33 (Figure 5).""When we compared phenotypic potential to mean growth rate, we surprisingly observed a weak positive correlation (R_squared=.079)". The reviewer notes that an R_squared of .079 corresponds to an R=0.28, so the second correlation is very similar to the first one, which was noted as very strong. Thus, I don't understand how the authors can make the claim that phenotypic potential is correlated to pleiotropy but not fitness. In general, the authors should report R rather than R_squared across the entire manuscript to enable more direct comparison of these correlations.

[5] In addition, as noted above, comparison to an independent study of DAmP allele fitness (Breslow et al) revealed an equally strong but opposite trend (alleles with high phenotypic potential tend to have fitness defects). This is a major issue that needs to be carefully addressed. More experiments need to be done to address the discrepancy with the Breslow results, especially since the correlation between phenotypic distance and the measurements from that dataset are closer to expectation. The data presented do not leave the reader with a clear perspective on this issue.

[6] In a similar vein, the authors did not see good agreement between their fitness data and the DAmP allele fitness measurements reported by Breslow et al. They attempt to discount the Breslow fitness data because the majority of alleles in the Breslow study do not exhibit fitness defects while their microcolony fitness assay identified more alleles with fitness defects. Thus, they imply that their data is high quality based on the assumption that DAmP alleles should result in loss-of-function phenotypes that translate to fitness defects. This is complete speculation given that it is unclear to what extent a DAmP allele reduces transcript stability and there are many factors that we expect would influence fitness of strains carrying these alleles including mRNA abundance, mRNA half-life, protein turn-over rates etc.

[7] Indeed, the authors also show that phenotypic potential is dependent on the type of genetic perturbation (DAmP vs. TET repression) and that the use of DAmP results in many false negatives (ie they missed many phenotypic stabilizers because the DAmP allele did not perturb essential gene function adequately). They should test more than 8 genes using an orthogonal system (TET off) to estimate their false negative rate and determine if the trends they observed would still hold true if a different genetic perturbation method was used.

[8] I think the following statement in the discussion is interesting, but I couldn't find the analysis/figure containing evidence supporting this-can the authors' include this? "A given strain's variability in nearly any morphological phenotype was correlated with its variability in most other

phenotypes. This was true despite the fact that mean values for these same traits were not correlated."

[9] The statement in the first paragraph of the discussion - "Most of these genes are poorly characterized because the field of yeast genetics has strongly relied upon gene knockout. For many of these genes our analysis represents one of the first steps in characterizing their functions" - is false. On the contrary, essential genes are the best characterized and most well studied genes in yeast.

1st Revision - authors' response 25 August 2014

Reviewer #1:

The authors show that the extent of cell morphological variability in yeast strains where essential genes are reduced in function using UTR insertions (DAmP alleles) does not correlate with the growth defect in each strain. In addition, using principle components analysis, they show that morphological variation tends to be correlated across 'phenotypes' i.e. that strains with stronger morphological defects tend to have multiple morphological defects. The basic dataset is interesting, but I think more could be got from it to raise the analysis to the level of MSB. In particular, I am not convinced that one of the main conclusions (phenotypic potential correlates (weakly) with phenotypic distance) is not trivial, given how these measures are calculated. Moreover the negative results (growth rate vs potential) would be firmer if more independent datasets were analyzed.

Comments

Pleiotropy. I think the use of pleiotropy in the title is somewhat misleading, given that here pleiotropy is defined from the morphological data. i.e. my interpretation is that there are typically pleiotropic morphological defects when morphology is affected, not that these 'correlate' with pleiotropy. There are many other ways to quantify pleiotropy from different data types e.g. from genetic interaction profiles (see e.g. Bellay et al. GR 2011) or from functional annotations or from physical interactions. The authors could investigate how these relate to morphological variability. However, I think the more straightforward conclusion is that cells with more severely affected morphological phenotypes tend to have more affected morphological phenotypes.

Sensitivity to measures used. The phenotypic potential measure is designed to capture the variability and magnitude of defects across many orthogonal morphological phenotypes. Is it really surprising therefore that strains in which phenotypes are strongly perturbed (distance measure) also have a higher potential? Especially given that this relationship is not exactly strong (see below). To quote the authors, 'This metric summarizes both the number of phenotype means that are affected by a gene's deletion as well as the magnitude of those effects, just as phenotypic potential summarizes the number and magnitude of effects on variation.' I would suggest the authors need to also present analyses that use alternative measures of both pleiotropy and potential that don't capture both number and magnitude so much: the number of traits affected is what is normally meant by pleiotropy, not how strongly each is affected.

RESPONSE:

The reviewers have provided important critiques of our measure for pleiotropy. In our original analysis we selected a measure of mean phenotypic change that would mirror phenotypic potential by capturing both the number and magnitude of individual changes. We felt that this would not lead to any trivial correlations since our measures of phenotypic variation were already corrected for any relationships between the standard deviations and means of the underlying phenotypes. We have amended the text to better emphasize this point; however, it is true that pleiotropy is traditionally viewed as a measure of the number of phenotypes altered regardless of the magnitudes of the changes. To address this, we have replaced phenotypic distance with a new measure. Pleiotropy is now measured by empirically defining confidence intervals around each phenotype in the wild type reference and then counting the number of phenotype means that differ

from this by more than one standard deviation for each mutant. We have also analyzed the available genetic interaction data for a subset of the DAmP strains but there was no relationship between number of genetic interactions and phenotypic potential.

Growth rate measurements. Growth rate datasets in yeast notoriously do not correlate well when compared across datasets/labs/techniques (see e.g. Baryshnikova et al. Nature methods 2010). How well do the fitness measures used here correlate (liquid vs colony assay etc)? In addition, microlony growth rates can underestimate population growth rates, so I think it important to use more than one dataset of bulk growth rates in the analyses before concluding a negative result. In addition, the Tet data also seems to potentially contradict the main conclusion (growth rate does seem related to potential when considered within or across the Tet strains) - this data should also be directly plotted (potential vs growth rate) and examined for each Tet strain and also across the strains.

RESPONSE:

This is an important point. Our measurements of growth rates for the DAmP strains differ dramatically from previously published data. We have now included data based on bulk population growth in liquid media. We have also extended our analysis of the Tet strains to include a more thorough comparison of growth rates and phenotypic potential at each condition.

Growth rate in yeast is very likely to be a fitness-related trait. However, morphology is not necessarily a fitness-related trait. Thus, I think the use of 'unfit' in the discussion is also misleading.

RESPONSE:

This has been changed

Comparison with the non-essential deletion collection data. The authors should re-analyze the deletion collection data with their new PCA-based analysis of morphological variation + a range of different datasets of growth rate. Is the lack of correlation with growth rate also upheld in the non-essential deletion collection data (after, for example, excluding deletions with no significant variation)? If not, why not?

RESPONSE:

It would be very informative to compare our dataset directly with the non-essential gene data, and we have made efforts to do this, but several factors preclude a satisfactory comparison. The most important reason is that the datasets are qualitatively different. The images were collected on different microscopes and at different magnifications with different depths of focus. The non-essential genes were also pressed between slides and cover slips while our preparations were centrifuged on glass-bottom plates for higher throughput. All of these differences have variable impacts on each phenotypic measure that CalMorph captures.

Fig 5. The correlation between phenotypic potential and mean phenotypic distance is not strong and seems to be largely driven by a small(ish) number of data points with distance >4. How strong is the correlation for the bulk of the dataset with distance <4? If strong, presenting this bulk data as a separate plot would be appropriate.

RESPONSE:

We have replaced our phenotypic distance measure with a more traditional count of the number of phenotypes altered. While our overall interpretation remains the same, the correlation remains strongest across the range from 0 to 5 phenotypes.

Fig 6. dead cell censoring. Removing the dead cells, do the 'low' phenotypic potential (more dead cells) and high phenotypic potential still differ in potential? i.e. are dead cells actually less variable

© European Molecular Biology Organization

in morphology (?).

RESPONSE:

Unfortunately, we do not have a practical method to remove all dead cells from the population before fixation nor is it possible to know precisely if a cell died before fixation. While this may be problematic for a few strains with high death rates, the vast majority of strains analyzed had death rates below 5%. Dead cells often suffer fragmentation of the cell wall or the nucleus and in these cases will not be analyzed by CalMorph.

abstract - delete 'novel system' (it is a previously used + described mutant collection)

RESPONSE:

This has been deleted

data availability - all of the cellular phenotype data, growth rates and rate variances analysed in this paper should be provided as data files in the supplement

RESPONSE:

We have now provided files that summarize our data as supplementary material. The image data are several terabytes and even the raw phenotypic data are several hundred megabytes in size and so will be provided as part of the Saccharomyces Morphological Database.

Intro 'However, an alternative hypothesis...'. To me, these two models do not seem mutually exclusive alternatives.

RESPONSE:

They are not mutually exclusive. The correction has been made.

Refs. Paaby A, Rockman M (2013) is incomplete; Lehner PlosG 2010 probably refers to PLosOne 2010 (?). Or Park/Lehner MSB 2013 (genetic hubs/capacitors have stable gene expression)?

RESPONSE:

Both references have been corrected.

"The flattening of the curve for distances greater than 4 is primarily due to a small number of mutants that have extremely large cell size and the fact that cell size is measured separately in each of the cell cycle categories." What's the data to support this statement?

RESPONSE:

This figure has been replaced so this is no longer relevant.

"Pleiotropy is, by far, the strongest correlate of phenotypic potential that we have identified. " Perhaps I have missed it, but please list all other correlates that were examined, either in supplement or simply in the text. 'by far' is also overselling the result, given the relatively weak correlation.

RESPONSE:

This is a fair point. We had looked for correlations with the number of protein-protein and

© European Molecular Biology Organization

genetic interactions and growth rates which have all previously been implicated as related to phenotypic variation. Our expanded analysis using the Tet system has lead to a more nuanced view of the relationship between growth rate and morphological variation and the text has been changed to reflect this.

The Anna Karenina principle - including the quotation would help the less literary reader (!)

RESPONSE:
Perhaps the first citation of Leo Tolstoy in this journal?

Figure axes should be more clearly labelled and indicate the units.

RESPONSE:
Corrected.

Reviewer #2:

In this manuscript, Chris Bauer and colleagues analyse the consequence of reducing the activity of yeast essential genes on the variability of cellular morphology. The study follows previous work of this lab on the effect of non-essential genes on this variability. Here, Bauer et al. used a collection of 873 DAmP mutants, where expression of the target gene is reduced by destabilization of the mRNA. They processed these mutants on automated analysis of fluorescence microscopy images and extracted measures of cell shape, size and DNA distribution pattern. They found that the majority of mutants display high phenotypic potential (a metric reflecting global cell-cell variability in several unrelated phenotypes), that this phenotypic destabilization was global (as opposed to specific to a few traits), that it correlated with pleiotropy (mutants with large alteration of mean trait values also display large variability) and, importantly, that it did not correlate with cell division rate (fitness).

The work is abundant and was thoroughly done. Remarkable qualities of the study are the large dataset (triplicates per strain), careful quality control to exclude unreliable phenotypic measures, the use of PCA to define independent traits, the validation of a subset of mutations effect by using a different type of gene silencing (doxycyclin-dependent transcrptional repression), and the single-cell resolution fitness assay wich allowed to disconnect cell-cell variability in morphology from cell-cell variability in growth rate (Fig6). The conclusion that essential genes act as capacitors comfort previous expectations (because these genes tend to be network hubs). But the absence of association between phenotypic destabilization and growth rate is novel and important. It also excludes the possibility that increased phenotypic potential merely reflects a general sickness of cells that are nearly dying because of the reduced activity of an essential gene.

The manuscript can nonetheless be improved by addressing the following points:

1. How does pleiotropy (of mean phenotypic values) varies along dox concentrations of the repressible strains? A dose effect on pleiotropy could reinforce or refine the correlation observed on the DAmP strains.

RESPONSE:
This is a great idea. We have performed the suggested analysis and have included a new figure in the manuscript. Although the range of phenotypic changes is reduced in the Tet dataset, the same relationship exists.

2. The raw single-cell data should be made available. This is very important to allow the community to reproduce the analysis and conclusions and to allow other future investigations of the data.

RESPONSE:

These entire data set and a subset of the raw and processed images will be made available in a searchable and downloadable form as part of the saccharomyces morphological database.

3. The subsection title "Robustness can be generalized across phenotype space" is confusing. Why not speaking about pleiotropy of phenotypic variability? This will increase consistency with the subsequent section "Pleiotropy is Strongly..." which refers to pleiotropy on mean phenotypic values.

RESPONSE:

This is an interesting perspective that we had not considered. We have added a sentence to draw the reader to the connection between the idea of pleiotropy of variability and what we call generalized robustness. However, since we are comparing magnitudes of variation in phenotypes, just as in our previous measure of phenotypic distance, pleiotropy may again be a misleading term.

4. Sentences "Moreover, there was no general... was -0.054" should be removed. It is not surprising: sd vs. mean dependence can be positive for some phenotypes and negative for others, or even non-monotonic. Seeing a global correlation near zero across all phenotypes does not add information on the degree of sd<->mean dependence for each trait.

RESPONSE:

Done.

5. A long paragraph of the discussion repeats the conclusions of the Richardson 2013 paper. This is too long and distracting. The study here focuses on buffering against environmental (stochastic) variation: it is confusing to spend lengthy text on the buffering against genetic variation. The distinction will be clearer if the paragraph is shortened.

RESPONSE:

Done.

6. Results of the PCA defining the phenotypes should be presented (histogram of variance explained per component, dot plots of strains along PC1,PC2...)

RESPONSE:

These figures have been included in the supplement.

7. The phenotypic potential values of DAmP strains (-0.77, 0.45; 0.85, 2.54, 1.99, 1.81) appear only in text. They should be printed or visualized on the figure to show the consistency between DAmP and the dox strains.

RESPONSE:

We have collected a completely new dataset for the Tet strains and due to changes in our microscopy capabilities, we feel it would be problematic to analyze all of the data together or to imply that a specific phenotypic potential value in one set is the same as in another. The main point is that several of the genes had very high values and the others did not.

8. Methods, page32: Normalization of loess residuals. It is not clear to me if the "variance of the variances" is the variance of the residuals.

RESPONSE:

This sentence has been clarified.

9. End of discussion: I agree that the results show that the "mutational target for modulation of phenotypic stability is large", but the authors should also discuss whether the mutational target is constrained or not: on one hand, it contains many essential genes, which do not evolve as freely as non-essential genes. On the other hand, the study shows that partial reduction of these genes' activity does not necessarily reduce fitness, at least in one environmental condition.

RESPONSE:
The discussion has been amended to discuss this point in more detail however, we can do little more than speculate.

10. Text on page 21 should refer to Figure 7.

11. Saying that "the field of yeast genetics has strongly relied upon gene knockout" is incorrect given the contributions of mid-XXth century. The authors probably meant "genomics" and not "genetics".

RESPONSE:
Corrected.

12.
- page 18: shape variation these traits: "of" these traits.
- Page 24. Ruled "out" and not "our"

RESPONSE:
Both typos have been changed.

Reviewer #3:

In this paper, Siegal and colleagues use HTP fluorescence microscopy to image 873 haploid yeast strains harboring so-called DAmP alleles of essential genes. The images were then analyzed using CalMorph (Yoshi Ohya's software) to measure 187 "phenotypes" that were reduced (using PCA) to a set of 41 phenotypes that explained more than 90% of total variance. The authors developed a "phenotypic potential" score to identify essential genes that act as general buffers of phenotypic variation. A phenotypic potential score is produced by calculating the average standard deviation based on the top 20 (out of 41) most variable phenotypes for each DAmP strain - after applying appropriate thresholds, the authors find between 20% of genes assayed (using a random permutation method) or 52% of genes assayed that exhibit high phenotypic potential and thus act as phenotypic stabilizers. This is significantly higher than for non-essential genes, showing that essential genes are enriched for
phenotypic stabilizers.
The authors then analyze their data to discover a strong positive correlation between phenotypic potential and pleiotropy, but not with fitness (using a micro-colony growth assay to measure fitness of 48 DAmPs with the greatest phenotypic potential scores and 48 DAmP alleles with the lowest phenotypic potential scores). A similar trend was not observed when comparing phenotypic potential to DAmP allele fitness measurements derived from an independent study (Breslow et al). Finally, 8 genes were selected that span a range of phenotypic potential scores and phenotypes for these genes were measured using strains carrying TET-repressible alleles of the genes - in this experiment, 6 of the genes showed the same high or low phenotypic potential seen with the DAmP allele strains, while 2 DAmP alleles with low potential exhibited much high phenotypic potential when assayed as TET alleles suggesting that DAmP alleles result in variable reduction of essential gene expression and that many
essential genes with high phenotypic potential may have been missed by screening the DAmP

© European Molecular Biology Organization

collection.
This is an interesting study, and the analysis of the phenotypes associated with DAmP allele strains using CalMorph should be a useful resource for the community. There are some serious issues with the paper as it now stands, though, which I outline below.

[1] The following statement is misleading: "Regardless of the phenotypic potential threshold we used, essential phenotypic stabilizers are highly enriched, relative to the set of all genes, for many GO terms related to RNA processing, ribosome biogenesis, transcription, phospholipid biosynthesis, and nuclear genome organization. However, there was not any statistically significant enrichment beyond that already present within the set of essential genes." In other words, there was no statistical enrichment among the set of genes tested which is not surprising given that essential genes are, in general, enriched for these features.

RESPONSE:

Well said. We have changed the text accrodingly.

[2] The authors claim that phenotypic stabilizers in their dataset tend to be pleiotropic - the strongest trend identified. I'm a little concerned that the metric used to measure pleiotropy is based on the same dataset used to measure phenotypic potential. This statement is unclear: "Moreover, there was no general underlying trend in the relationship between mean and variation within our data set to begin with". If this is true, why was loess used to begin with.

RESPONSE:
Please see response to reviewer #1.

[3] I am also concerned about whether the sampling error on the mean for a given strain and phenotype is directly proportional to the variance in that phenotype for that strain. It's possible that sampling error, when averaged across a large number of phenotypes, may partially explain the observed correlation between the phenotypic distance and phenotypic potential measurements (Fig. 5). Can the authors rule this out?

RESPONSE:

The sampling error of the variance is larger than the sampling error of the mean so any error in our measurement of mean phenotypes is the lesser or two evils. Given that at least 1000 cells were analyzed for each genotype we do not believe sampling error can explain our results. Far fewer cells were analyzed in yeast deletion collection and yet the number of phenotypic stabilizers identified was smaller in that data set despite a larger sampling error.

[4] The authors conclude that phenotypic potential is correlated to pleiotropy but not correlated with fitness - a conclusion they state in their title. The contrast between the conclusions made in these statements is striking given that they're supported by similar statistics. For example, "A plot of phenotypic distance versus phenotypic potential reveals a strong positive relationship with Spearman's rho (R) = 0.33 (Figure 5).""When we compared phenotypic potential to mean growth rate, we surprisingly observed a weak positive correlation (R_squared=.079)". The reviewer notes that an R_squared of .079 corresponds to an R=0.28, so the second correlation is very similar to the first one, which was noted as very strong. Thus, I don't understand how the authors can make the claim that phenotypic potential is correlated to pleiotropy but not fitness. In general, the authors should report R rather than R_squared across the entire manuscript to enable more direct comparison of

these correlations.

RESPONSE:

This is a valid criticism. We have conducted a more thorough analysis with the tet strains and have moderated our conclusions. We have also changed all R squared references to simply R.

[5] In addition, as noted above, comparison to an independent study of DAmP allele fitness (Breslow et al) revealed an equally strong but opposite trend (alleles with high phenotypic potential tend to have fitness defects). This is a major issue that needs to be carefully addressed. More experiments need to be done to address the discrepancy with the Breslow results, especially since the correlation between phenotypic distance and the measurements from that dataset are closer to expectation. The data presented do not leave the reader with a clear perspective on this issue.

RESPONSE:

See response to #6 below.

[6] In a similar vein, the authors did not see good agreement between their fitness data and the DAmP allele fitness measurements reported by Breslow et al. They attempt to discount the Breslow fitness data because the majority of alleles in the Breslow study do not exhibit fitness defects while their microcolony fitness assay identified more alleles with fitness defects. Thus, they imply that their data is high quality based on the assumption that DAmP alleles should result in loss-of-function phenotypes that translate to fitness defects. This is complete speculation given that it is unclear to what extent a DAmP allele reduces transcript stability and there are many factors that we expect would influence fitness of strains carrying these alleles including mRNA abundance, mRNA half-life, protein turn-over rates etc.

RESPONSE:

For reasons unrelated to this study, we were specifically interested in the ~150 DAmP alleles that had growth rates reported as greater than wild type. We collected the 94 strains with the highest reported growth rates and found that, in our hands, all 94 of them showed growth defects ranging from mild to very severe based on bulk OD measurements. This was our initial reason for suspicion of the growth rate data from Breslow et al. We now have included an independent set of bulk growth rates that correlate much better with our microcolony assay data than with the original published figures based on competition assays. We have no way to explain the inconsistency, but we remain confident that most of the DAmP strains exhibit measurable growth defects.

[7] Indeed, the authors also show that phenotypic potential is dependent on the type of genetic perturbation (DAmP vs. TET repression) and that the use of DAmP results in many false negatives (ie they missed many phenotypic stabilizers because the DAmP allele did not perturb essential gene function adequately). They should test more than 8 genes using an orthogonal system (TET off) to estimate their false negative rate and determine if the trends they observed would still hold true if a different genetic perturbation method was used.

RESPONSE:

The analysis of Tet repressible strains has been expanded to address this point.

© European Molecular Biology Organization

[8] I think the following statement in the discussion is interesting, but I couldn't find the analysis/figure containing evidence supporting this-can the authors' include this? "A given strain's variability in nearly any morphological phenotype was correlated with its variability in most other phenotypes. This was true despite the fact that mean values for these same traits were not correlated."

RESPONSE:

The statement regarding variance correlations is supported by figure 4. Figure S5 has now been included in the supplement to show the general lack of correlations between mean phenotypes.

[9] The statement in the first paragraph of the discussion - "Most of these genes are poorly characterized because the field of yeast genetics has strongly relied upon gene knockout. For many of these genes our analysis represents one of the first steps in characterizing their functions" - is false. On the contrary, essential genes are the best characterized and most well studied genes in yeast.

RESPONSE:

This point is well taken and we have changed the text to reflect this.

2nd Editorial Decision 16 October 2014

Thank you again for submitting your work to Molecular Systems Biology. We have now heard back from the two referees who accepted to evaluate the revised study. As you will see, the referees are now globally supportive and we are pleased to inform you that we will be able to accept you study for publication pending the following points:

- please address the remaining minor point raised by the reviewers.
- the complete imaging and phenotypic datasets should be made public. Please include a 'data availability' section at the end of Materials & Methods that indicate where the datasets can be dowloaded. If the SCMD is not yet ready, we would suggest to deposit the entire dataset to Dryad and include the respective DOI in the data availability section.

Thank you for submitting this paper to Molecular Systems Biology.

Reviewer #1:

The data analysis is now more rigorous, however the abstract now says almost nothing - it does not even report the main results and conclusions!

Reviewer #2:

The points I raised have been properly addressed in the revisions. There is one error in text (section results): Supp Figure 11 shows a correlation between phen pot and pleiotropy for 0 to 4 phenotypes affected, not 0 to 5.

2nd Revision - authors' response 17 November 2014

The remained concerns of reviewers have been addressed. The most important changes are highlighted below:

- The abstract has been updated to better reflect changes to the manuscript.
- References to figure S11 have been changed
- The original data and images have been made public through dryad doi:10.5061/dryad.ft7dj.

© European Molecular Biology Organization
